# Influence of soil nutrients on the presence and distribution of CPR bacteria in a long-term crop rotation experiment

**DOI:** 10.3389/fmicb.2023.1114548

**Published:** 2023-07-27

**Authors:** Alinne L. R. Santana-Pereira, Francesco S. Moen, Beatrice Severance, Mark R. Liles

**Affiliations:** Department of Biological Sciences, Auburn University, Auburn, AL, United States

**Keywords:** candidate phyla radiation, metagenome-assembled genomes, metabolism, phylogenetic diversity, soil ecology

## Abstract

Bacteria affiliated with the Candidate Phyla Radiation (CPR) are a hyper-diverse group of ultra-small bacteria with versatile yet sparse metabolisms. However, most insights into this group come from a surprisingly small number of environments, and recovery of CPR bacteria from soils has been hindered due to their extremely low abundance within complex microbial assemblages. In this study we enriched soil samples from 14 different soil fertility treatments for ultra-small (<0.45 μm) bacteria in order to study rare soil CPR. 42 samples were sequenced, enabling the reconstruction of 27 quality CPR metagenome-assembled genomes (MAGs) further classified as Parcubacteria/Paceibacteria, Saccharibacteria/Saccharimonadia and ABY1, in addition to representative genomes from Gemmatimonadetes, Dependentiae and Chlamydae phyla. These genomes were fully annotated and used to reconstruct the CPR community across all 14 plots. Additionally, for five of these plots, the entire microbiota was reconstructed using 16S amplification, showing that specific soil CPR may form symbiotic relationships with a varied and circumstantial range of hosts. Cullars CPR had a prevalence of enzymes predicted to degrade plant-derived carbohydrates, which suggests they have a role in plant biomass degradation. Parcubacteria appear to be more apt at microfauna necromass degradation. Cullars Saccharibacteria and a Parcubacteria group were shown to carry a possible aerotolerance mechanism coupled with potential for aerobic respiration, which appear to be a unique adaptation to the oxic soil environment. Reconstruction of CPR communities across treatment plots showed that they were not impacted by changes in nutrient levels or microbiota composition, being only impacted by extreme conditions, causing some CPR to dominate the community. These findings corroborate the understanding that soil-dwelling CPR bacteria have a very broad symbiont range and have metabolic capabilities associated to soil environments which allows them to scavenge resources and form resilient communities. The contributions of these microbial dark matter species to soil ecology and plant interactions will be of significant interest in future studies.

## 1. Introduction

Since the discovery of Microgenomatia (OD11) from the Obsidian pools in Yellowstone National Park (Hugenholtz et al., 1998), the CPR have been reported in diverse environments leading to the discovery of Parcubacteria (OD1) and Asconditabacteria (SR1) taxa (Harris et al., 2004). The systematic study of these uncultured bacteria led to the description of the hyper-diverse monophyletic group described as the CPR (Brown et al., 2015), comprising more than 20% of known bacterial diversity (Hug et al., 2016; Parks et al., 2017), significantly increasing the previously described Patescibacteria clade (Rinke et al., 2013).

Members of this group have small cells (0.009 ± 0.002 μm^3^) (Luef et al., 2015) and patchy metabolisms with incomplete or lacking major pathways such as nucleotide, amino acid and lipid metabolism, TCA cycle or electron transport chains (Castelle et al., 2018), and are predicted to rely on alternate mechanisms to generate and exploit the proton motor force (Wrighton et al., 2012; Kantor et al., 2013). Despite these observed genome reductions, CPR bacteria have innovative features such as alternate fermentation enzymes (Kantor et al., 2013), hybrid RuBisCOs that function in an archaeal-like fashion (Wrighton et al., 2016), and deployment of other archaeal-like enzymes (Ghuneim et al., 2018). Additionally, they have been predicted to participate in nitrogen, sulfur and carbon cycling (Wrighton et al., 2014; Danczak et al., 2017). The foundation of our current understanding of CPR taxa focused on aquifers, groundwater, and associated sediment (Wrighton et al., 2012; Castelle et al., 2013; Brown et al., 2015; Luef et al., 2015; Anantharaman et al., 2016; Danczak et al., 2017). Therefore, the study of these ultra-small bacteria in other environmental contexts is imperative to better understand the extent of their diversity, metabolic capabilities, and ecological roles.

Soil environments harbor enormous bacterial diversity (Torsvik and Ovreas, 2002); however, members of the CPR group have only sparsely been reported in soil microbiome surveys (Yeates et al., 2000; Wagner et al., 2009; Lemos et al., 2020; Santana-Pereira et al., 2020). Recovery of soil dwelling CPR is challenging due to their extremely low abundances (Herrmann et al., 2019) and intronic 16S rRNA genes that evade amplicon-based surveys (Brown et al., 2015). Despite the advent of genome-resolved metagenomics (Sharon and Banfield, 2013), the challenges in assembling sequences from complex soil metagenomes has hindered the reconstruction of these low abundant CPR genomes from soil and to date only a few studies have achieved this goal (Kroeger et al., 2018; Woodcroft et al., 2018; Lemos et al., 2020; Sharrar et al., 2020; Nicolas et al., 2021).

Another significant challenge in accessing CPR genomes is their low relative abundance within soil microbiota. Other studies have used size fractionation to isolate distinct populations of microbial cells (Portillo et al., 2013), and to enrich for novel phylogenetic groups including CPR bacteria that may not be observed from studies of bulk soil (Nakai, 2020; Lee et al., 2021; Nicolas et al., 2021). We therefore used an ultra-small (<0.45 μm) bacterial cell enrichment protocol to enrich for low abundance CPR bacteria, and other members of the rare biosphere, from soil samples of the Cullars Rotation (Auburn, AL). The Cullars Rotation is the oldest ongoing soil fertility experiment in the southern United States, with a 3-year rotation of corn, cotton, soybean and winter legumes across 14 plots that vary significantly in NPK fertilizer and lime amendments (Zhao et al., 2015). Direct sequencing of the small cell-derived metagenomic DNA allowed the reconstruction of 31 MAGs predominantly from CPR taxa. CPR genomes were annotated for their predicted potential for biomass degradation, and their abundance across Cullars Rotation soil treatment groups was correlated with differences in nutrient, pH, and metal levels, shedding light into their possible ecological roles and distribution patterns in soil.

## 2. Materials and methods

### 2.1. Sampling site

**Figure 1:**
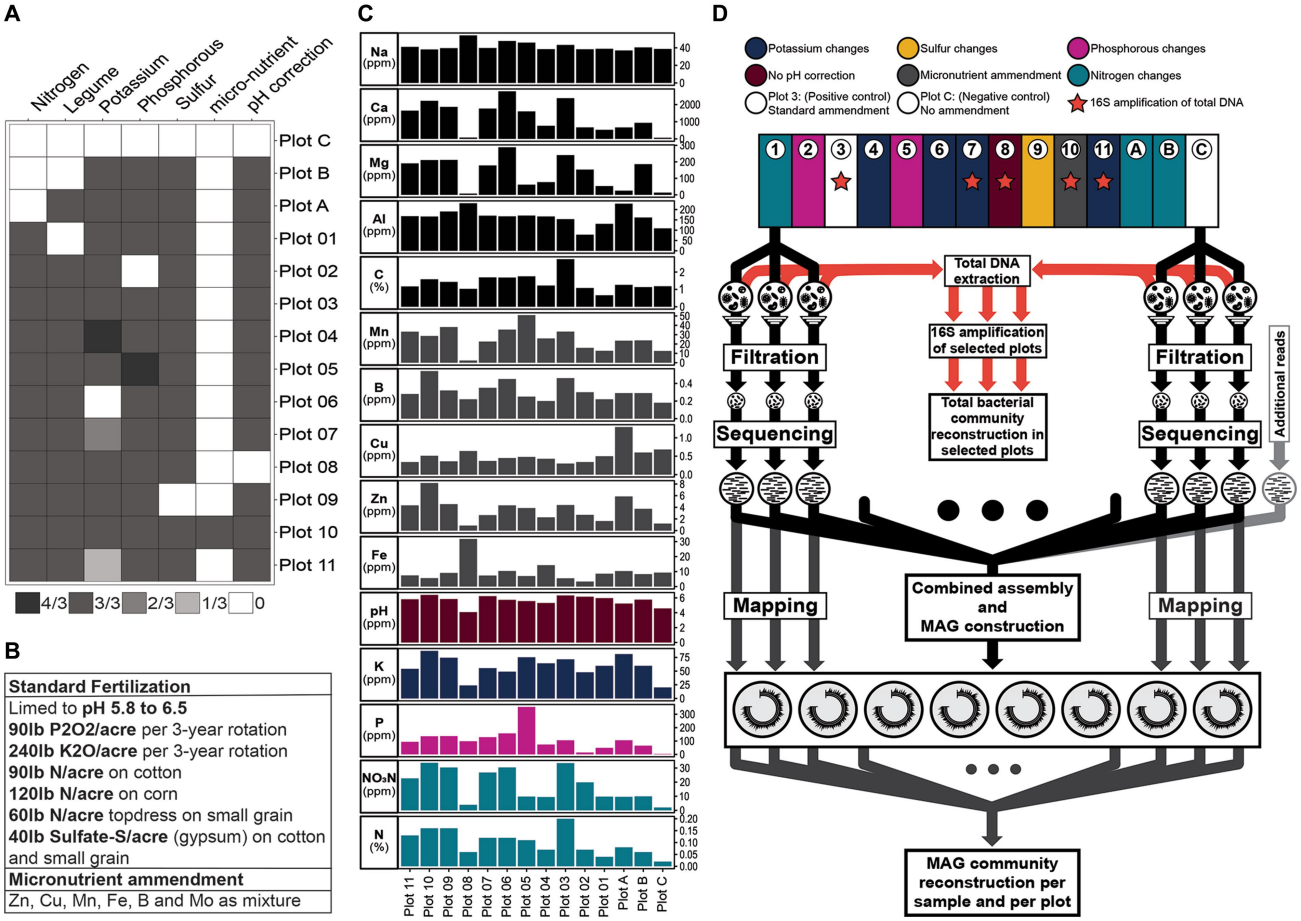
Representation of Cullars Rotation plots and Fertilization treatments. **(A)**. Schematic representation of Cullars Rotation Plot layout and Soil treatments administered. pH correction: pH correction with lime. Fractions represent the amount added to the plot with Standard Fertilization as reference (3/3). **(B)**. Table with the Standard Fertilization treatments applied to each plot (Mitchell et al., 2005). **(C)**. Average soil nutrient parameters from sampled bulk soil. **(D)**. Overview of the methods employed to reconstruct the MAGs from the filtration enrichment (mini-metagenome), reconstruct the MAG community and reconstruct the total bacterial community using 16S amplification from soil total DNA extraction.

The Cullars Rotation is a century old crop rotation experiment alternating between five crops during the course of a year (cotton, crimson clover, corn, wheat and soybean) (Mitchell et al., 2011). The rotation is composed of 14 plots for the study of the effects of controlled deficiencies of phosphorus (P), potassium (K), nitrogen (N) and sulfur (S), as well as other soil amendments such as lime (for pH control) and addition of a micronutrient mix containing boron (B), zinc (Zn), manganese (Mn), copper (Cu) and iron (Fe) on crop yield (Mitchell et al., 2005) (Figure 1).

### 2.2. Sample collection and soil analysis

Soil samples were obtained from the Cullars Rotation in March 2019. Three topsoil samples from each one of the 14 plots in Cullars Rotation were collected for soil DNA extraction, alongside ~400 g of bulk soil for analysis. The samples for DNA extraction were immediately transported to the lab and kept refrigerated at 4°C until processing. Processing of the 42 soil samples (3 replicates/14 plots) were completed on the same day as sampling. The bulk soil samples were submitted for analysis at Auburn University’s Soil Testing Laboratory the same day. Soil nutrients were obtained using Mehlich I Extraction and analyzed by ICP. Measurements were obtained for calcium (Ca), K, magnesium (Mg), P, aluminum (Al), B, Cu, Fe, Mn, sodium (Na), Zn, Nitrate-Nitrogen (NO_3_-N), all in parts per million (ppm). Percent carbon ©, N, and pH were also measured.

### 2.3. Ultra-small cell enrichment

For each sample, 5 g of soil was suspended in 25 mL of 0.2% tetrasodium pyrophosphate buffer (pH 8.0), which has been shown to dissociate microbial cells from soil particulates (e.g., clay, sand and silt) while retaining the microbial community structure associated with bulk soil (Portillo et al., 2013; Yuan et al., 2018). Suspensions were homogenized for 10 min, with gentle inversions, followed by 10 cycles of intermittent vortexing for 5 s. The soil slurry was subjected to a low-speed centrifugation (5,000 x *g* for 3 min) to precipitate larger soil particulates and eukaryotic cells. The supernatant was collected and serially filtered through a 5.0 μm cellulose membrane, then 1.2 μm and 0.45 μm SUPOR® membranes (Pall Corporation, Port Washington, New York) in tandem. The <0.45 μm fraction was then pelleted by centrifugation (20,000 x *g* for 20 min) and DNA was isolated from the cell pellet using the Power Soil DNA isolation kit (MoBIO Labs) to obtain the <0.45 μm fraction metagenomic DNA (Figure 1). There were three topsoil replicates from each of the 14 plots in the Cullars Rotation, resulting in 42 different samples from which a < 0.45 μm fraction metagenome was generated.

### 2.4. Shotgun sequencing, binning, and genome annotation

Each <0.45 μm fraction metagenome was uniquely indexed and paired-end sequenced using an Illumina HiSeq platform in one dedicated run (Figure 1). Raw reads from all metagenomes were processed using Trimmomatic v0.39 (Bolger et al., 2014). The remaining reads from all 42 samples were combined and assembled with MEGAHIT v1.2.9 (Li et al., 2015). The reads from each sample were mapped back against the assembled contigs using BWA v0.7.17 (Li and Durbin, 2009). Parallel binning was conducted with MetaBAT2 v2.15 (Kang et al., 2019), MaxBin 2.0 v2.2.7 (Wu et al., 2016) and CONCOCT v1.1.0 (Alneberg et al., 2014). Resulting genomic bins were curated using DasTool v1.1.4 (Sieber et al., 2018). The completeness and contamination of each bin was assessed using CheckM v1.1.3 (Parks et al., 2015) using the standard bacterial marker set and a CPR-specific phylogenetic marker set (Brown et al., 2015). Bins with completeness below 65%, contamination above 10% or strain heterogeneity above 10% were dropped from subsequent analyses. Bins with completeness above 85%, contamination below 5% and strain heterogeneity below 5% were considered high-quality MAGs, while bins with completeness above 75%, contamination below 5% and strain heterogeneity below 5% were considered good-quality MAGs and the remainder were considered draft MAGs. To aid in genome assembly and binning, an additional set of reads from a similar sequencing run of a pilot enrichment for Cullars Rotation’s Plot C was added to the read pool for a second round of assembly and binning following the same steps and criteria above. The total bins obtained were dereplicated using Derep v0.12.8 (Olm et al., 2017) and used in the subsequent analysis.

### 2.5. MAG phylogenetic classification

MAGs were taxonomically classified using two strategies: 16S rRNA gene sequence homology and phylogenetic marker gene voting. First, 16S rRNA sequences were extracted using CheckM v1.1.3 (Parks et al., 2015) and a custom package for CPR 16S rRNA extraction and intron removal (Brown et al., 2015). The MAG-associated 16S rRNA genes were classified against the SILVA database (Quast et al., 2012) (accessed February, 2020) using SINA (Pruesse et al., 2012) through the web client. A homology threshold of 75% was used for phylum classification and 80% for class classification. For the second approach, 13 phylogenetically informative marker genes (Supplementary Table S1) corresponding to ribosomal proteins with similar phylogenetic signal to 16S rRNA (Gregor et al., 2016), were extracted from each bin using CheckM v1.1.3. The markers were annotated against the nr/nt NCBI database. Then each marker “casts a vote” according to their taxonomic annotation. The taxonomy with the higher number of “votes” was assigned to the MAG. Finally, the consensus classification from the two strategies was applied to the MAG. Both strategies used NCBI taxonomy, which is itself based on the taxonomy and nomenclature proposed by Brown et al. (2015).

MAGs classified as CPR were selected for a robust additional classification by constructing a concatenated maximum likelihood tree. A curated collection of reference CPR genomes was constructed by downloading 1,200 CPR genomes from NCBI (accessed October 2019) and filtering out low quality MAGs with the program CheckM v1.1.3 by the following requirements: >75% completeness, < 5% contamination and strain heterogeneity. The set of 13 phylogenetic markers was extracted from each reference genome. For each marker, the Cullars MAG sequences and the references sequences were aligned using MAFFT v7.475 (Katoh and Standley, 2013), trimmed with TrimAl v1.4.1 (Capella-Gutierrez et al., 2009). Then, the marker alignments were concatenated using Phylemon 2.0 (Sanchez et al., 2011) and manually inspected with Geneious 10.3.^1^ The concatenated alignment of 13 markers was used to generate a maximum likelihood tree with 1,000 bootstraps using RAxML v8.2.9 (Stamatakis, 2014). Marker genes from the Dependentiae MAGs were used to root the tree. The final tree was visualized and annotated using iTOL (Letunic and Bork, 2019), using Brown et al. (2015) proposed nomenclature. In an effort to also comply with the efforts toward an standardized bacterial classification and taxonomy (Parks et al., 2020), our MAGs were classified according to GTDB (Parks et al., 2022) using GTDB-Tk v2.2 (Chaumeil et al., 2022), and utilized the (Brown et al., 2015) proposed nomenclature for phylogenetic affiliations.

### 2.6. MAG metabolic potential analysis

To assess metabolic and nutrient utilization potential for each of our CPR MAGs, each of the sufficient quality MAGs and members of the same taxa from the curated collection of reference genomes, were annotated using DRAM v1.3.5 (Shaffer et al., 2020). This software utilizes a series of databases (UniRef90, PFAM, dbCAN, Refseq viral, VOGDB, and MEROPS peptidase) to annotate genes and reconstruct the organism’s metabolic profile. DRAM annotations were analyzed with special attention to carbohydrate utilization *via* carbohydrate-active enzymes (CAZymes) (Lombard et al., 2014). Profiles between MAGs and their corresponding references were compared using a 2-way analysis of variance (ANOVA) based on a linear regression model using the lme4 (Bates et al., 2015) and emmeans (Lenth et al., 2019) packages. Output was analyzed in RStudio v. 4.0.3.

### 2.7. Cullars <0.45 μm fraction bacterial community reconstruction and distribution across Cullars plots

The <0.45 μm fraction bacterial community from Cullars soil was reconstructed from the assembled MAGs. To estimate MAG counts, reads from each sample were individually mapped back against each MAG using Bowtie2 (Langmead and Salzberg, 2012) with high sensitivity. Well mapped reads were counted using SAMTools v1.3 (Li et al., 2009). The matrix with read counts per sample was imported into Rstudio. Library sizes were normalized using the cumulative scale sum strategy (CSS) with metagenomeSeq (Paulson et al., 2013). Counts were then normalized by genome length by dividing the total read length by the MAG genome size (bp). Coverages below 1% were discarded as inconclusive and assigned to zero (Levy-Booth et al., 2021). Fractions were rounded up to generate a list of normalized natural counts. These counts were used to reconstruct MAG community and calculate average MAG relative abundance per plot.

Rarefaction curves for each sample were calculated using the package Ranacapa (Kandlikar et al., 2018). For both MAG– and 16S rRNA gene-based community assessments, non-metric multidimensional scaling (NMDS) and correlation analysis (CA) were performed with Bray-Curtis calculated distances using the package PhyloSeq (McMurdie and Holmes, 2013). Differences in community composition across plots were accessed by performing PERMANOVA with 999 permutations and subsequent multivariate homogeneity of groups dispersions (Betadisper) using the package Vegan (Oksanen et al., 2013). The same package was used to perform vector fitting and surface fitting of Cullars Rotation soil parameters with significant correlation to community changes (*p* < 0.05).

### 2.8. Bulk soil bacterial community reconstruction and distribution across Cullars rotation plots

Total DNA was extracted from bulk soil samples which were the same used for CPR cell enrichment. DNA from plots 03, 08, 10 and 11 were used for 16S rRNA gene PCR amplification. The V4 region of the 16S rRNA gene was amplified and paired-end sequenced. Paired-end reads were processed using FLASH (Magoc and Salzberg, 2011) to produce raw tags, which were quality filtered following the QIIME2 protocol (Caporaso et al., 2010). The clean high-quality tags were compared against the SILVA database and chimeras were removed using UCHIME (Edgar et al., 2011). Sequences with >97% similarity were clustered into the same operational taxonomic unit (OTU) using Uparse. Taxonomic annotation of OTU representative sequences was carried out against the SILVA SSU database using Mothur (Schloss et al., 2009).

OTU absolute abundance was normalized by rarefaction, and the subsequent counts were used for alpha and beta diversity analyses. The community comparisons between plots and ordinations were constructed using the same programs outlines above for the MAG communities. Linear discriminant analysis Effect Size (LEfSE) analysis was conducted using the LEfSe software (Segata et al., 2011).

### 2.9. Network analysis

Normalized counts from the 10 most abundant phyla in the16S and MAG abundance data were combined to access the possible relationships between the relative abundance of CPR MAGs and 16S ribotypes from soil bacteria. The resulting count table was used to build the network using the package SPIEC-EASI (Kurtz et al., 2015). The graphs were drawn using the package iGraph (Csardi and Nepusz, 2006) and exported into Cytoscape (Smoot et al., 2011) for visualization.

## 3. Results

### 3.1. Recovering ultra-small bacteria genomes from Cullars rotation soil

A previous study of a Cullars Rotation soil metagenome revealed that CPR bacteria were present in this soil in extremely low abundance, but were accessible using a direct cloning and sequencing approach that avoided PCR and 16S rRNA gene-associated biases (Santana-Pereira et al., 2020). Therefore, for each of three samples from the 14 plots at Cullars Rotation, ultra-small cells <0.45 μm were enriched from soil samples to directly sequence and obtain CPR genome bins. From the 124 non-replicated bins, a total of 42 genomic bins were curated ranging from 400 Kb to 3.5 Mb (average 1.17 Mb). We identified 27 MAGs with acceptable quality, which was defined as completeness below 65%, contamination over 10% or strain heterogeneity over 10% (Supplementary Figure S1). These MAGs had an average completeness of 86.8%, with 5 bins later identified as CPR with 100% completeness, and an average genome size of 1.13 Mb in accordance with the reduced genome size characteristic of CPR bacteria. A noticeable exception was MAG DT 16 with a genome size of 3.55 Mb and completeness of 78.6%; however, this relatively larger genome was affiliated with the phylum Gemmatimonadetes which is not known to have a small size but has been previously observed to be capable of transmission through a small sized filter (Portillo et al., 2013).

### 3.2. Taxonomical classification of Cullars MAGs

CPR classification has experienced several revisions (Rinke et al., 2013; Brown et al., 2015; Parks et al., 2017, 2018), highlighting the challenges associated with conducting phylogenetic analyses of uncultured microorganisms (Konstantinidis et al., 2017). Initial studies on CPR taxonomy, based on up ta few thousands of genomes, categorized it as a monophyletic group encompassing several individual candidate phyla, such as Saccharibacteria, and the superphyla Parcubacteria and Microgenomates, each containing numerous candidate phyla (Brown et al., 2015; Anantharaman et al., 2016; Hug et al., 2016). This classification is most congruent to NCBI taxonomy and thus popularized. With the increasing availability of MAGs and genomes, much more comprehensive studies using from 8 to more than 90 thousand genomes made widespread rank normalization and standardization possible, settling on Patescibacteria/CPR as a single phylum (Parks et al., 2017, 2018). This framework was used to develop the Genome Taxonomy DataBase (GTDB) (Chaumeil et al., 2019; Parks et al., 2020), which proposed new nomenclatures and taxonomic ranks for CPR. Thus the previous candidate phyla Saccharibacteria, Parcubacteria and ABY1, correspond to the classes Saccharimonadia, Paceibacteria and ABY1, respectively (Supplementary Table S2). Due to their widespread use and congruence with popular NCBI nomenclature, the names CPR, Saccharibacteria and Parcubacteria will be favored throughout the paper.

**Figure 2:**
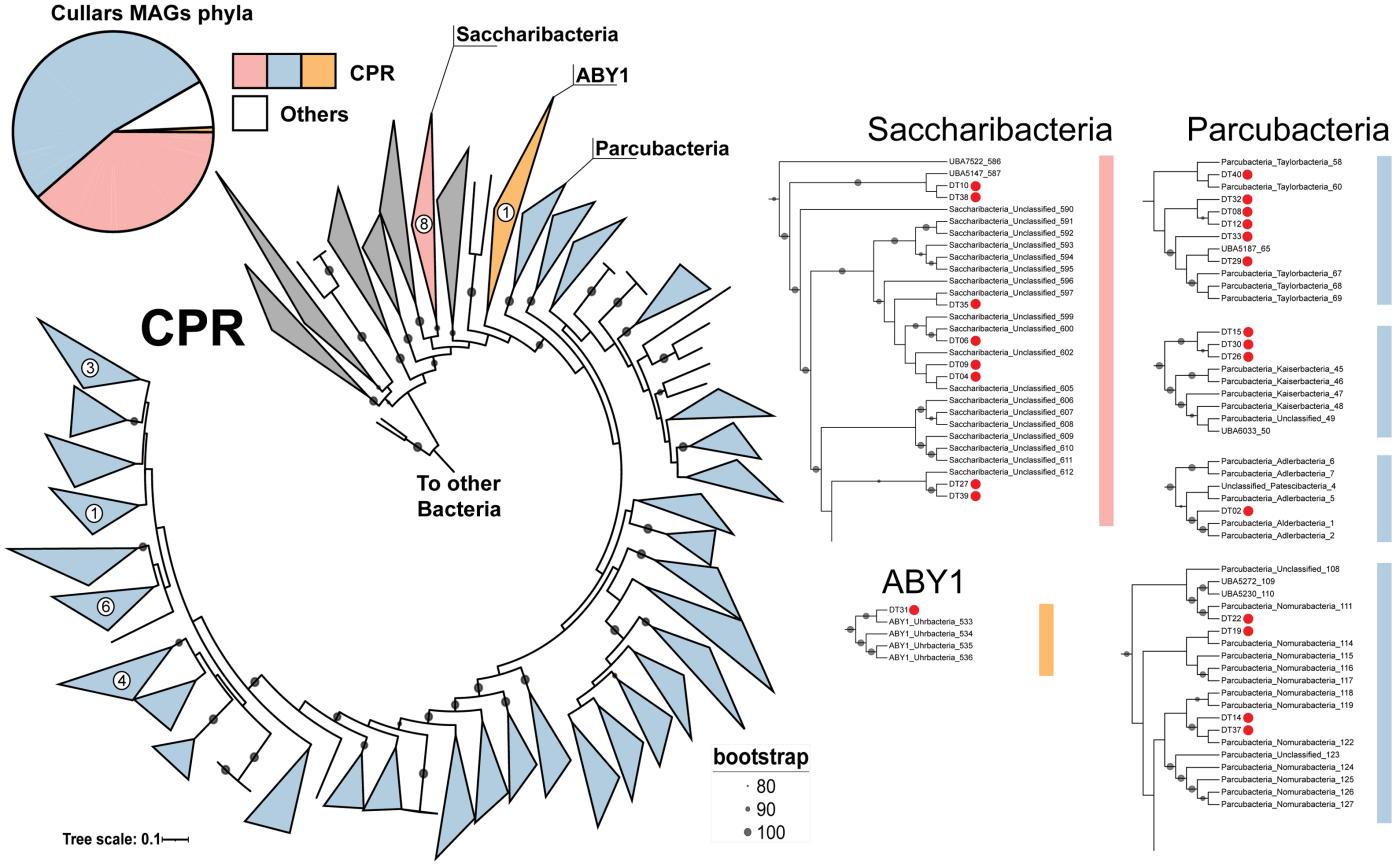
Classification of all Cullars MAGs and the proportion of each phyla. *Total MAGs does not include MAGs filtered out by the completeness, strain heterogeneity and contamination thresholds. Concatenated maximum likelihood tree of 13 phylogenetic marker genes from Cullars CPR and 920 reference genomes allowed further classification of CPR MAGs. MAGs were classified as Saccharibacteria (Saccharimonadia), ABY1 and Parcubacteria (Paceibacteria). The Parcubacteria group is expanded in the tree due to its intrinsic diversity. Zoomed in regions offers context to MAGs placement. Red dots denote Cullars MAGs.

A collection of 13 phylogenetically informative marker genes (Supplementary Table S1) and 16S rRNA genes were extracted from Cullars MAGs and were independently compared to the NCBI GenBank nr/nt and SILVA databases, respectively, to form a consensus homology classification of the MAGs. All curated MAGs were classified, with one representative MAG recovered from each of the phyla Actinobacteria, Chlamydiae, Dependentiae and Gemmatimonadetes, and the remaining belonging to the CPR group. Within CPR, there were representatives of the Parcubacteria (16), Saccharibacteria (10) and ABY1 (1) groups (Figure 2). Additionally, CPR MAGs were classified using the GTDB classifier, showing high congruence with the previous classification, since Parcubacteria, Saccharibacteria and ABY1 MAGs, were classified as Paceibacteria, Saccharimonadia and ABY1, respectively. Moreover, GTDB provided finer classification up to family level. Interestingly, a number of MAGs associated with underrepresented taxa in GTDB were recovered from Cullars Soil, such as representatives of families VEQN01 and CAYRIW01, which have only 3 and 1 reference genomes, respectively.

Given the high degree of diversity among members of the CPR group, the candidate CPR MAGs were further classified by constructing a concatenated maximum likelihood tree using 13 phylogenetic markers and 920 reference genomes, allowing the selection of closest relatives for metabolic potential comparisons (Figure 2). The tree confirmed the previous taxonomical assignments. Cullars CPR MAGs affiliated with the Parcubacteria/Paceibacteria class, were all from the order UBA9983_A and distributed across 10 families that correlate to the broad groups Nomurabacteria, Taylorbacteria, Adlerbacteria and Kaiserbacteria in NCBI taxonomy. Additionally, two MAGs (DT 10 and DT 38) clustered with an unclassified CPR MAG to form a deeper branch within Saccharibacteria (Figure 2). These MAGs were classified by GTDB as belonging to the order UBA4664, which carries only 9 references, corresponding to 1.25% of all Saccharibacteria genomes in the database. Indeed, this low representation, whether due to sampling or environmental rarity, might explain the observed deeper branching, although it might be mitigated with the insertion of more reference genomes. The ABY1 group MAG was further classified into the Uhrbacteria group.

### 3.3. Metabolic potential of soil dwelling CPR

**Figure 3:**
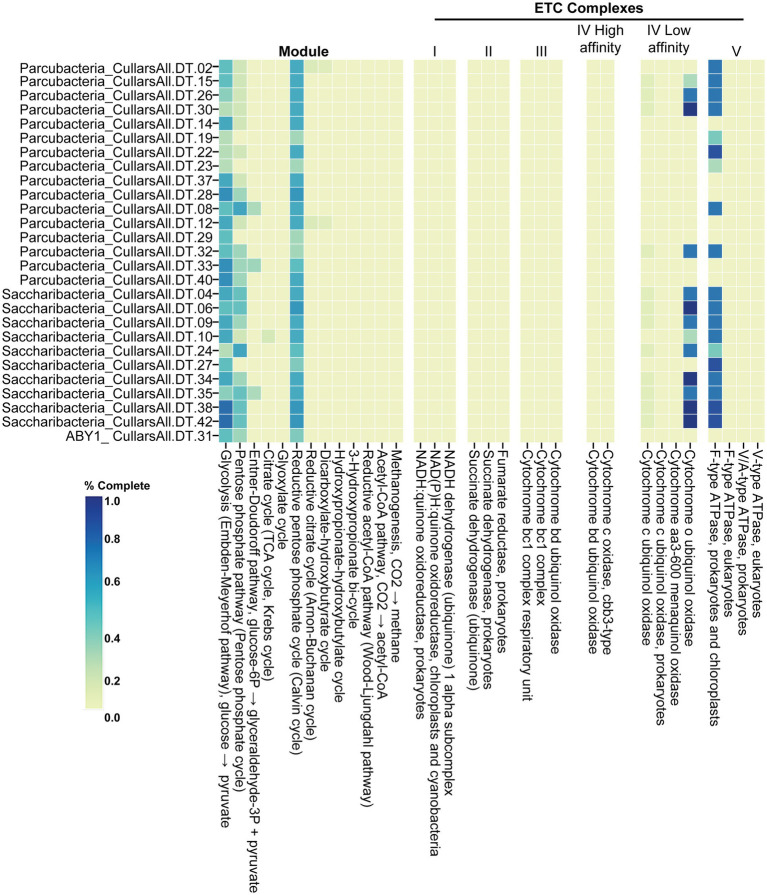
Energetic metabolic potential of Cullars MAGs. % Complete: level of completeness of the pathway, measured as presence of genes coding for necessary enzymes to carry out each step.

The MAGs recovered from Cullars Rotation soil represent three distinct CPR classes including the Saccharibacteria (10), Parcubacteria (16), and ABY1 (1). These MAG genomes (Figure 3) were compared to a curated collection of 124 genome references from NCBI (Saccharibacteria, 43; Parcubacteria, 49; ABY1, 32) to assess their metabolic potential utilizing the metabolic profiling tool DRAM (Supplementary Figure S2).

ABY1, Saccharibacteria, and Parcubacteria MAGs carried partially complete glycolysis, pentose phosphate and reductive pentose phosphate pathways, as consistently observed among these classes of CPR. Additionally, they were found to have nearly complete F-type ATPase. All MAGs were lacking a complete tricarboxylic acid cycle (TCA), subunits of nicotinamide adenine dinucleotide (NADH) dehydrogenase and electron transport chain (ETC), which indicates an anaerobic and fermentative metabolism, although the presence of complete or nearly complete F-type ATPases was widespread. Interestingly, Parcubacteria, namely from the family UBA2103, and Saccharibacteria from Cullars were more likely to carry mostly complete operons coding for cytochrome o ubiquitol oxidase. Cytochrome o functions by catalyzing the oxidation of ubiquinol (CoQH_2_) into ubiquinone (CoQ), coupled with the reduction of O_2_ into water, supplying energy to pump H+ into the outer membrane contributing to the formation of a proton motive force (Aussel et al., 2014). Interestingly, it was more common among the soil CPR than the references.

**Figure 4:**
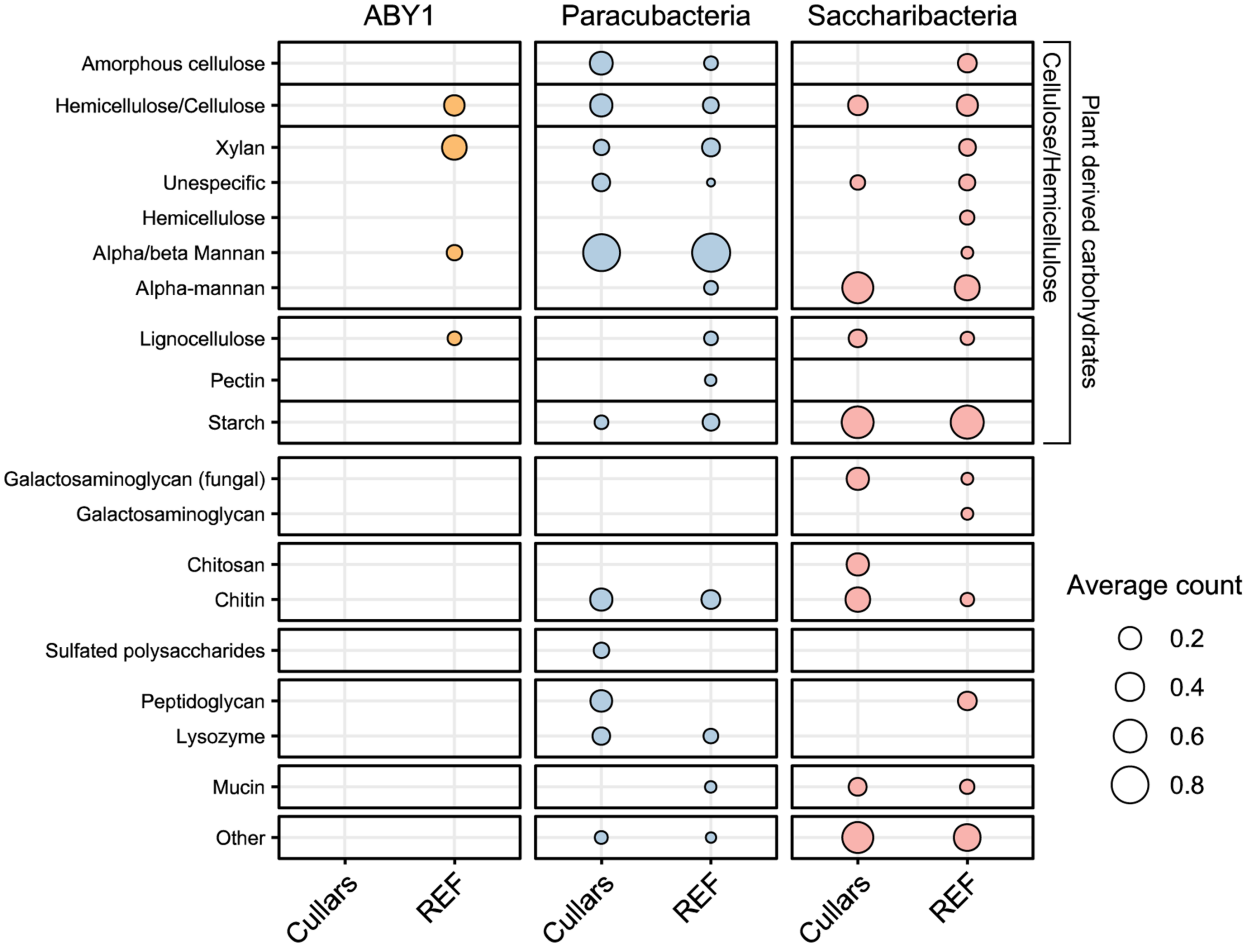
Carbohydrate degradation potential of Cullars CPR MAGs and their references. Hits correspond to GH genes identified against the CAZy database, which was also used to obtain substract information. Circles represent the averaged hits for references and Cullars MAGs per substrate category.

The CAZyme database and its enzyme classes were utilized to understand Cullars MAGs carbohydrate metabolic potential, namely glycoside hydrolases (GH, carbohydrate degradation), glycotransferases (GT, carbohydrate biosynthesis), carbohydrate esterases (CE, hydrolysis of carbohydrate esters), carbohydrate-binding modules (CBM, adhesion of carbohydrates), and auxiliary activities (AA, redox coenzymes for lignocellulose degradation) (Supplementary Figure S4). Most Cullars MAGs contained genes for enzymes associated with starch, cellulose/hemicellulose, and chitin degradation with the top hits among the Saccharibacteria and Parcubacteria, but not present in the Cullars ABY1 MAG (Figure 4). Interestingly, only Cullars Saccharibacteria carried the potential for lignin degradation. This continues to support soil CPR as containing the necessary enzymes for degradation of complex plant carbohydrates and soil necromass including bacterial and fungal cell walls.

ABY1 and Saccharibacteria carbon substrate metabolic profiles were not found to be statistically different from that of the reference genomes available on NCBI (*p* > 0.05). However, Cullars Parcubacteria MAGs did demonstrate a strongly statistically difference from their references (*p* < 0.001). Parcubacteria was the most represented CPR in our MAGs and particular samples (i.e., DT.28) contained several hits for particular enzyme classes for peptidoglycan and chitin degradation, suggesting these soil microorganisms may be particularly adept for scavenging dead or decaying soil microflora.

Cullars CPR MAGs did not demonstrate any ability to participate in nitrogen or methane cycling. Select Saccharibacteria were predicted to have some capacity for dissimilatory and assimilatory sulfate reduction. Cullars MAGs were predicted to degrade organic forms of nitrogen, particularly, the amino acid methionine in the cysteine biosynthesis pathway. Likewise, Cullars CPR had many predicted peptidases, indicating the ability to degrade peptides from the soil environment.

### 3.4. Distribution of the ultra-small bacteria in Cullars rotation soil

**Figure 5:**
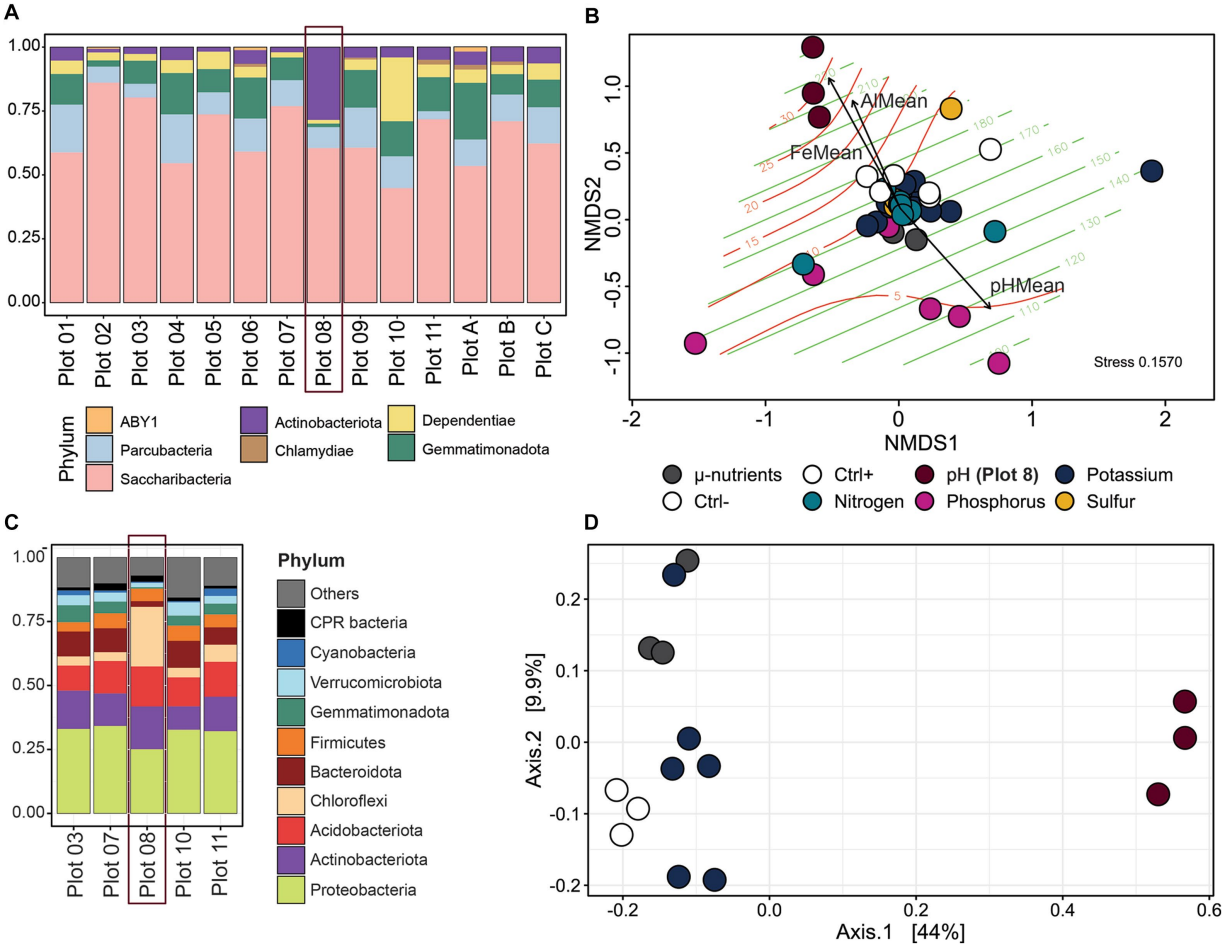
**(A)** MAG community reconstruction from Cullars soil at different plots corresponding MAG average relative abundance, ranked by Phyla. **(B)** NMDS ordination of all sample communities with Bray-Curtis distances. Colors emphasize the varying nutrients and pH on the plots. Ctrl +: Plot 3 (Standard Fertilization); Ctrl -: Plot C (No fertilization or amendment). Green lines: Aluminum levels; Red lines: Iron levels. Vectors are soil parameters significantly related to community changes (p < 0.05). Emphasis on Plot 8 samples, which are pulled in the direction of the highest Fe and Al concentrations. **(C)** Overall community reconstruction from Cullars soil of selected Cullars plots using 16S amplification. **(D)** Ordination plot by PCA of all sample communities with Bray-Curtis distances.

Sampling the Cullars Rotation soil fertility plots offers a unique opportunity to assess the influence of nutrient availability and soil parameters on the soil CPR community, due to its century-old controlled nutrient deficiency experiments (Mitchell et al., 2011) (Figure 1). To reconstruct the MAG community in each plot, reads from each sample were mapped back against the curated MAGs and normalized to account for variations in library size and genome length (Figure 1). The normalized, filtered counts were used to reconstruct the communities in each of the plots in Cullars Rotation (Figure 5A). Additionally, soil nutrient content for each plot was also measured creating a profile that could be correlated with changes in community structure (Figure 5B).

MAG communities are composed predominantly of Saccharibacteria, followed by Gemmatimonadetes and Dependentiae, even though the latter two have only one representative MAG each. Despite having the higher and most diverse number of representatives, Parcubacteria accounted for a significantly smaller proportion of the community, indicating that taxa dominance was observed in the sampled metagenomes. Interestingly, there was an observed spike in Actinobacteria abundance in Plot 08, which received no pH correction treatment and had a significantly lower pH than other treatment groups.

MAG abundance within the different CPR taxa varied among the different experimental treatment groups (Supplementary Figure S5). In particular, Plot 08 (no pH correction) showed the greatest shift in community structure, with 78% of the relative abundance corresponding to one Actinobacteria and two Saccharibacteria bins, which were dominant exclusively in this plot, while also having the highest number of recovered Parcubacteria genomes. Dependentiae and Gemmatimonadetes genomes were also recovered across all plots, with the later peaking in abundance under increased phosphorous amendment (Plot 05) and dropping to marginal abundance when there was no soil pH correction (Plot 08) indicating a susceptibility to very acidic soils. Otherwise, the abundance of CPR genomes was not significantly different among the different Cullars Rotation plots.

NMDS ordination was used to visualize communities, dissimilarities in relation to fertilization treatment. To further explore the role of soil parameters in community compositions, the measurements of soil macro and micro-nutrients were modeled to the community distance matrix (Figure 5B). Community profiles did vary significantly across plots (PERMANOVA, *p* = 0.001 and Betadisper, *p* = 0.8537) with the soil plots with an acidic pH having the most dramatic effect in clustering (*p* = 0.015). The lack of pH correction coupled with the addition of fertilization treatment resulted in the most acidic pH across all plots for Plot 08 (pH = 4.08 ± 0.08), surpassing even Plot C which received no amendment nor fertilization (pH = 4.58 ± 0.04); however, pH alone could not account for the observed differences (PERMANOVA, *p* = 0.199), with similar trends not observed for Plot C, which also has a very acidic soil. The environmental fit analysis reveals that the factors with the strongest correlation to the changes in the community were aluminum (Al) and iron (Fe) concentrations (*p* = 0.001 and *p* = 0.0001, respectively).

### 3.5. Overall microbiota community and network analysis

Next, the relationship between the MAG community and overall bacterial community was explored. 16S rRNA gene amplicon data were used to reconstruct the overall bacterial community, recovering representatives of 40 phyla. The overall community was dominated by Proteobacteria, Actinobacteria, Acidobacteria, Bacteroidota, and Firmicutes (Figure 5C). Plot 08 had lower alpha diversity (Supplementary Figure S6) and an overrepresentation of the phylum Chloroflexi. Communities between plots were significantly different (*p* = 0.001), with Plot 08 also clustering away from other plots in PCA ordinations (Figure 5D). Similarly to the MAG community shifts, an acidic pH was correlated with significant 16S community differences (*p* = 0.004); however, the only soil parameter with a significant correlation to the community was Al concentration (*p* = 0.001).

Linear discriminant analysis effect size (LEfSe) was used to identify taxa highly correlated to community shifts in each plot that can act as biomarkers. 18 OTUs were marked as Plot 08 biomarkers at varying taxonomical levels, with the strongest signal belonging to the phylum Chloroflexi, class Ktedonobacteria (Supplementary Figure S7).

**Figure 6:**
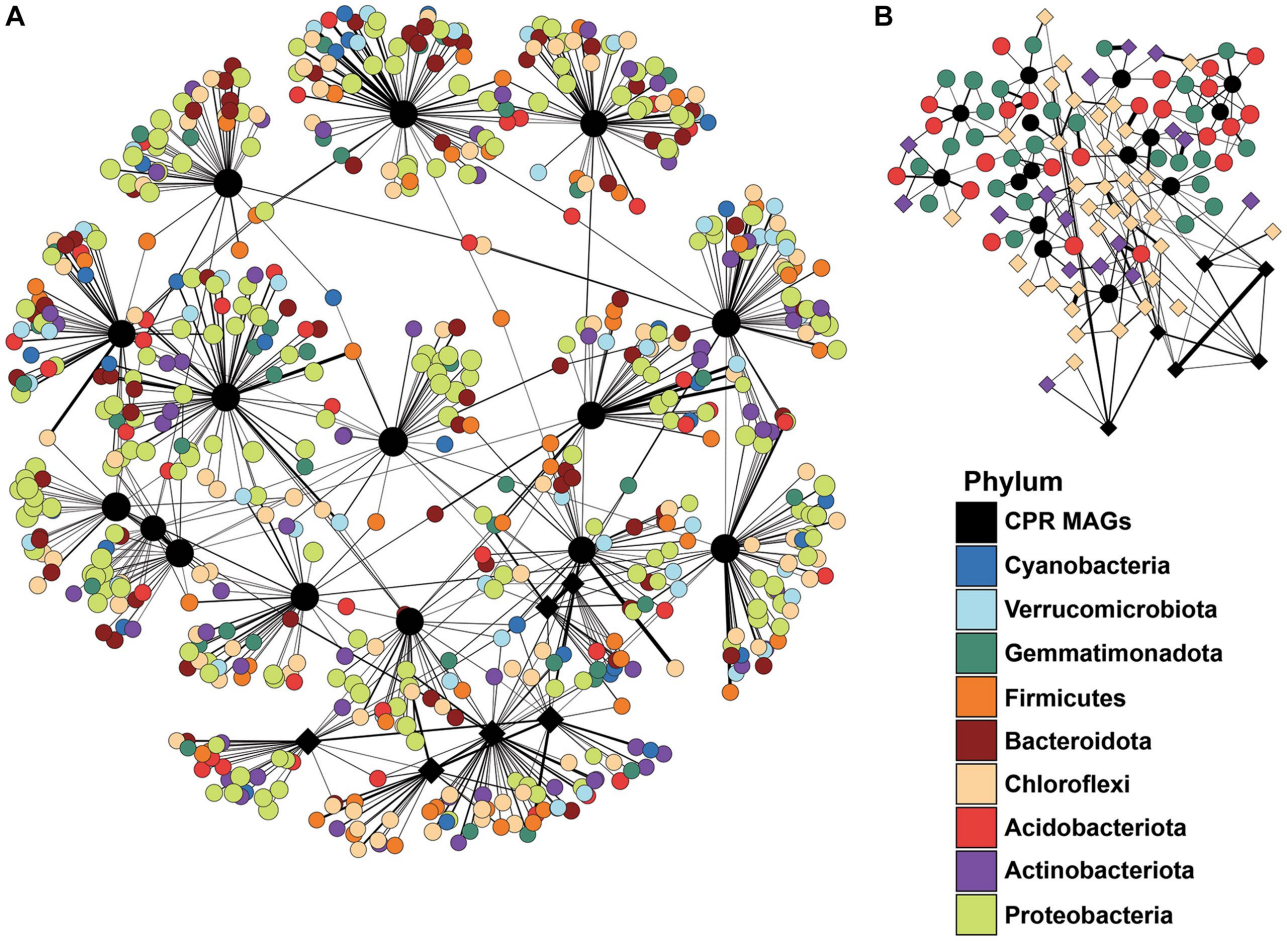
**(A)** Network analysis filtered by all the OTUS belonging to the main phyla from the overall microbiota that interacts positively with the Cullars MAGs. Line thickness corresponds to correlation weight **(B)** Network analysis filtered by all the OTUS belonging to the plot biomarkers (defined by LEfS analysis) that interacts positively with the Cullars MAGs. Diamond shaped nodes: Prominent MAGs and biomarkers in Plot 08.

The potential relationship between Cullars’ MAGs and members of the soil microbiome was explored by constructing networks between Cullars’ MAGS and soil phyla (Figure 6). The MAGs were correlated with the occurrence of diverse phyla, albeit the weight of the edges was low. No particularly strong relationship was identified between the MAGs or any particular taxa in the overall soil microbiome, even when focusing on OTUs and MAGs distinctively associated with Plot 08, such as MAGs DT35 and DT24, or Plot 08-specific biomarkers such as Chloroflexi or Acidobacteria taxa.

## 4. Discussion

Most studies of ultra-small bacteria have centered around the CPR phylum due to their unusual metabolic potential (Jaffe et al., 2020; Chiriac et al., 2022) and astounding phylogenetic diversity (Hug et al., 2016; He et al., 2021). Despite their notable presence in groundwater, aquifers and associated sediment (Luef et al., 2015; Anantharaman et al., 2016; Chiriac et al., 2022), it was indicated that aquatic CPR are mobilized from soils (Herrmann et al., 2019). Hence soil became a focus of CPR study, supported by the reconstruction of CPR genomes from Amazonian (Kroeger et al., 2018) and Antarctic soils (Woodcroft et al., 2018), via deep-sequencing.

Following the observation of CPR in Cullars rotation soil in very low abundances (Santana-Pereira et al., 2020), we employed an enrichment protocol targeting the small fraction (< 0.45 μm) of the microbiota for the recovery of soil CPR with much modest sequencing resources then the otherwise terabyte efforts previously reported (Anantharaman et al., 2016; Woodcroft et al., 2018), although consideration of possible biases and incomplete sampling must be taken into account. This approach allowed the study of 14 different cultivated plots under different fertilization regimens, offering a unique opportunity to observe soil CPR communities under controlled soil nutrient variation. This study demonstrated that CPR are widespread in Cullars Rotation soils, and potentially in the soil environment generally, albeit in low abundance. Genome reduction (<1.5Mbp) and sparse metabolisms were also observed in Cullars Rotation soil-derived CPR genomes, pointing toward symbiotic life-styles, as previously observed (Brown et al., 2015; Castelle et al., 2018; Herrmann et al., 2019).

Cullars CPR had similar metabolic restrictions as their aquatic counterparts: incomplete nucleotide, amino-acid and lipid metabolisms. The lack of an TCA cycle and ETC elements, suggest that soil CPR are mainly fermentative, akin to previously characterized CPR (Castelle et al., 2018). However, while those CPR were associated with anoxic environments, Cullars CPR were collected from an aerobic fraction of the topsoil (Castelle et al., 2017). Interestingly, soil Saccharibacteria and UBA2103 (Parcubacteria/Paceibacteria) were more likely to carry a Cytochrome o complex, suggesting the ability to reduce O_2_ to H_2_O. Although uncommon, the presence of ETC components have been described in CPR and associated with O_2_ scavenging instead of energy production (Brown et al., 2015). Indeed, Coenzyme Q_8_ (CoQ_8_) plays an important role in oxidative stress by neutralizing reactive oxygen species (ROS) (Aussel et al., 2014), therefore it is possible that the combined action of CoQ_8_ and Cytochrome o confers aerotolerance to soil CPR by scavenging ROS and O_2_ simultaneously *via* CoQ_8_ redox cycling (ubiquinone ↔ ubiquinol). However, the simultaneous presence of Cytochrome o and ATPases, suggest that Cullars Saccharibacteria and UBA2103 (Parcubacteria/Paceibacteria) carry some capacity for respiration, which has been previously observed and hypothesized in other CPR recovered from soil (Nicolas et al., 2021). Although, no NADH or succinate dehydrogenases were identified in Cullars CPR, ROS scavenging *via* ubiquinol formation could be a source of electrons for Cytochrome o and proton motor force creation. It is noteworthy that most MAGs carried ATPases, even in the absence Cytochrome o, or any other ETC component, suggesting that they might be used in yet unknown ways.

CPR bacteria have been predicted to ferment complex substrates (Kantor et al., 2013), and Cullars Saccharibacteria and Parcubacteria were particularly adept at degrading a variety of carbohydrates including plant-derived sugars (Starr et al., 2018; Taubert et al., 2019), suggesting their participation in plant necromass turn over and Carbon cycling in the soil. However, due to their patchy fermentation pathways, it is unclear if these organisms preferentially derive their energy from direct biomass degradation or from scavenging processed derivatives (Taubert et al., 2019; Figueroa-Gonzalez et al., 2020).

Despite different selective pressures in aquatic environments and soils, the sugar degradation profiles of Cullars CPR and references were surprisingly similar, with the retention of plant-matter degradation enzymes by the later, thus corroborating the observation that aquatic CPR are mobilized from soils (Herrmann et al., 2019). On the other hand, Cullars Parcubacteria displayed distinct enrichment of chitin and peptidoglycan degradation enzymes and cytochrome o complexes, suggesting soil specific adaptations toward scavenging dead or decaying soil microfauna under oxic conditions.

While some CPR have been shown to live an endosymbiotic lifestyle (Gong et al., 2014; Bor et al., 2018), we did not find specific correlations between CPR MAGs and specific bacterial taxa, which points toward reliance on available bacterial cohorts in each plot for the supply of the needed building blocks (Anantharaman et al., 2016), perhaps forming episymbiotic relationships with diverse hosts (He et al., 2021), instead of forming strict host-specific interactions. Unsurprisingly, the mini-metagenome approach identified other symbiotic bacteria within these soils including environmental Chlamydia and Dependentiae (TM6), which are other examples of bacterial lifestyles that result in genome reduction (McCutcheon and Moran, 2011).

The Cullars Rotation soil fertility experiment offered a unique resource to observe how the CPR community responds to controlled nutrient deficiencies in soil. Although they were not likely directly used by CPR, they have great influence in plant matter input and microbiota activity (Zhao et al., 2015). Yet, nutrient availability changes did not influence CPR communities, indicating that their particular niches are not disturbed by the observed fluctuations in the overall microbiota composition and activity, nor by nutrient bioavailability and crop growth. Moreover the lack of taxa-specific trends in CPR distribution across the plots and the ability of all Cullars CPR to withstand and grow under diverse conditions suggests that soil CPR have dynamic ecological roles (Jaffe et al., 2020).

CPR communities, and the overall microbiota, were the most affected by a sharp decrease in pH caused by lack of pH correction resulting in heavy metal solubilization and potential toxicity, leading to decreased crop yields (Mitchell et al., 2005), microbiota diversity and activity (Zhao et al., 2015). Despite that, some Cullars CPR, especially two particular Saccharibacteria MAGs, thrived and outcompeted others, dominating the community. Although CPR have been observed in extreme conditions, usually Microgenomates and Parcubacteria (Harris et al., 2004; Gagen et al., 2020; Hosokawa et al., 2021), Saccharibacteria are not commonly associated with these environments.

More extensive sampling could have the potential to uncover more CPR diversity, since rarefaction curves show that most of the plots were under-sampled. Indeed, Cullars CPR sequences previously captured in a soil metagenomic library (Santana-Pereira et al., 2020) were not observed in this study. More stringent filtering (Nicolas et al., 2021) or increased sequencing depth could be implemented in future studies to better MAG construction and thus diversity recovery.

Nevertheless, these findings offer valuable insight into the soil dwelling CPR community and their possible ecological niches. We have shown that CPR are rare but widespread in soils, despite differences in nutrient availability and microbiota composition. Although soil CPR share many of the metabolic limitations of groundwater or sediment-associated CPR (Jaffe et al., 2020; Lemos et al., 2020), there is a notable bias toward degradation of plant-derived matter and even the development of specific adaptations of oxic environments, including the potential for a unique respiration pathway coupled with ROS scavenging. By observing the CPR communities in parallel, we were able to verify that Cullars CPR do not form strict symbiotic relationships, nor depend on specific hosts for survival, behaving like scavenging ectosymbionts. Cullars CPR appear to live in the fringes of the microbiota undisturbed due to their low abundances and resourceful metabolisms, allowing them to thrive under adverse conditions. Future studies should explore the contributions of soil CPR bacteria to important biogeochemical processes and especially plant-microbial interactions.

## Data availability statement

The datasets presented in this study can be found in online repositories. The names of the repository/repositories and accession number(s) can be found at: https://www.ncbi.nlm.nih.gov/genbank/, BioProject accession number PRJNA901447.

## Author contributions

ML conceived of the research and secured funding for this project. AS-P and FM conducted the research experiments and analysed the results. AS-P, FM, and BS conducted the bioinformatic analyses. AS-P was primarily responsible for writing the first draft and all authors edited and approved the manuscript for submission. All authors contributed to the article and approved the submitted version.

## Funding

This publication was supported by the Alabama Agricultural Experiment Station and the Hatch program of the National Institute of Food and Agriculture, U.S. Department of Agriculture, Hatch project # ALA021-1-13733.

## Conflict of interest

The authors declare that the research was conducted in the absence of any commercial or financial relationships that could be construed as a potential conflict of interest.

## Publisher’s note

All claims expressed in this article are solely those of the authors and do not necessarily represent those of their affiliated organizations, or those of the publisher, the editors and the reviewers. Any product that may be evaluated in this article, or claim that may be made by its manufacturer, is not guaranteed or endorsed by the publisher.

## Footnotes

^1^ https://www.geneious.com

## References

[ref1] Alneberg J. Bjarnason B. S. de Bruijn I. Schirmer M. Quick J. Ijaz U. Z.. (2014). Binning metagenomic contigs by coverage and composition. Nat. Methods 11, 1144–1146. doi: 10.1038/nmeth.3103, PMID: 25218180

[ref2] Anantharaman K. Brown C. T. Hug L. A. Sharon I. Castelle C. J. Probst A. J.. (2016). Thousands of microbial genomes shed light on interconnected biogeochemical processes in an aquifer system. Nat. Commun. 7:13219. doi: 10.1038/ncomms13219, PMID: 27774985 PMC5079060

[ref3] Aussel L. Pierrel F. Loiseau L. Lombard M. Fontecave M. Barras F. (2014). Biosynthesis and physiology of coenzyme Q in bacteria. Biochim. Biophys. Acta 1837, 1004–1011. doi: 10.1016/j.bbabio.2014.01.015, PMID: 24480387

[ref4] Bates D. Mächler M. Bolker B. Walker S. (2015). Fitting linear mixed-effects models Usinglme4. J. Stat. Softw. 67:i01. doi: 10.18637/jss.v067.i01, PMID: 37415114

[ref5] Bolger A. M. Lohse M. Usadel B. (2014). Trimmomatic: a flexible trimmer for Illumina sequence data. Bioinformatics 30, 2114–2120. doi: 10.1093/bioinformatics/btu170, PMID: 24695404 PMC4103590

[ref6] Bor B. McLean J. S. Foster K. R. Cen L. To T. T. Serrato-Guillen A.. (2018). Rapid evolution of decreased host susceptibility drives a stable relationship between ultrasmall parasite TM7x and its bacterial host. Proc. Natl. Acad. Sci. U. S. A. 115, 12277–12282. doi: 10.1073/pnas.1810625115, PMID: 30442671 PMC6275545

[ref7] Brown C. T. Hug L. A. Thomas B. C. Sharon I. Castelle C. J. Singh A.. (2015). Unusual biology across a group comprising more than 15% of domain bacteria. Nature 523, 208–211. doi: 10.1038/nature14486, PMID: 26083755

[ref8] Capella-Gutierrez S. Silla-Martinez J. M. Gabaldon T. (2009). trimAl: a tool for automated alignment trimming in large-scale phylogenetic analyses. Bioinformatics 25, 1972–1973. doi: 10.1093/bioinformatics/btp348, PMID: 19505945 PMC2712344

[ref9] Caporaso J. G. Kuczynski J. Stombaugh J. Bittinger K. Bushman F. D. Costello E. K.. (2010). QIIME allows analysis of high-throughput community sequencing data. Nat. Methods 7, 335–336. doi: 10.1038/nmeth.f.303, PMID: 20383131 PMC3156573

[ref10] Castelle C. J. Brown C. T. Anantharaman K. Probst A. J. Huang R. H. Banfield J. F. (2018). Biosynthetic capacity, metabolic variety and unusual biology in the CPR and DPANN radiations. Nat. Rev. Microbiol. 16, 629–645. doi: 10.1038/s41579-018-0076-2, PMID: 30181663

[ref11] Castelle C. J. Brown C. T. Thomas B. C. Williams K. H. Banfield J. F. (2017). Unusual respiratory capacity and nitrogen metabolism in a Parcubacterium (OD1) of the candidate phyla radiation. Sci. Rep. 7:40101. doi: 10.1038/srep40101, PMID: 28067254 PMC5220378

[ref12] Castelle C. J. Hug L. A. Wrighton K. C. Thomas B. C. Williams K. H. Wu D.. (2013). Extraordinary phylogenetic diversity and metabolic versatility in aquifer sediment. Nat. Commun. 4:2120. doi: 10.1038/ncomms3120, PMID: 23979677 PMC3903129

[ref13] Chaumeil P. A. Mussig A. J. Hugenholtz P. Parks D. H. (2019). GTDB-Tk: a toolkit to classify genomes with the genome taxonomy database. Bioinformatics 36, 1925–1927. doi: 10.1093/bioinformatics/btz848, PMID: 31730192 PMC7703759

[ref14] Chaumeil P. A. Mussig A. J. Hugenholtz P. Parks D. H. (2022). GTDB-Tk v2: memory friendly classification with the genome taxonomy database. Bioinformatics 38, 5315–5316. doi: 10.1093/bioinformatics/btac672, PMID: 36218463 PMC9710552

[ref15] Chiriac M. C. Bulzu P. A. Andrei A. S. Okazaki Y. Nakano S. I. Haber M.. (2022). Ecogenomics sheds light on diverse lifestyle strategies in freshwater CPR. Microbiome 10:84. doi: 10.1186/s40168-022-01274-3, PMID: 35659305 PMC9166423

[ref16] Csardi G. Nepusz T. (2006). The igraph software package for complex network research. InterJ complex syst 1695, 1–9.

[ref17] Danczak R. E. Johnston M. D. Kenah C. Slattery M. Wrighton K. C. Wilkins M. J. (2017). Members of the candidate phyla radiation are functionally differentiated by carbon– and nitrogen-cycling capabilities. Microbiome 5:112. doi: 10.1186/s40168-017-0331-1, PMID: 28865481 PMC5581439

[ref18] Edgar R. C. Haas B. J. Clemente J. C. Quince C. Knight R. (2011). UCHIME improves sensitivity and speed of chimera detection. Bioinformatics 27, 2194–2200. doi: 10.1093/bioinformatics/btr381, PMID: 21700674 PMC3150044

[ref19] Figueroa-Gonzalez P. A. Bornemann T. L. V. Adam P. S. Plewka J. Révész F. von Hagen C. A.. (2020). Saccharibacteria as organic carbon sinks in hydrocarbon-Fueled communities. Front. Microbiol. 11:587782. doi: 10.3389/fmicb.2020.587782, PMID: 33424787 PMC7786006

[ref20] Gagen E. J. Levett A. Paz A. Bostelmann H. Valadares R. B. S. Bitencourt J. A. P.. (2020). Accelerating microbial iron cycling promotes re-cementation of surface crusts in iron ore regions. Microb. Biotechnol. 13, 1960–1971. doi: 10.1111/1751-7915.13646, PMID: 32812342 PMC7533318

[ref21] Ghuneim L. J. Jones D. L. Golyshin P. N. Golyshina O. V. (2018). Nano-sized and filterable bacteria and archaea: biodiversity and function. Front. Microbiol. 9:1971. doi: 10.3389/fmicb.2018.01971, PMID: 30186275 PMC6110929

[ref22] Gong J. Qing Y. Guo X. Warren A. (2014). "Candidatus Sonnebornia yantaiensis", a member of candidate division OD1, as intracellular bacteria of the ciliated protist *Paramecium bursaria* (Ciliophora, Oligohymenophorea). Syst. Appl. Microbiol. 37, 35–41. doi: 10.1016/j.syapm.2013.08.007, PMID: 24231291

[ref23] Gregor I. Droge J. Schirmer M. Quince C. McHardy A. C. (2016). PhyloPythiaS+: a self-training method for the rapid reconstruction of low-ranking taxonomic bins from metagenomes. PeerJ 4:e1603. doi: 10.7717/peerj.1603, PMID: 26870609 PMC4748697

[ref24] Harris J. K. Kelley S. T. Pace N. R. (2004). New perspective on uncultured bacterial phylogenetic division OP11. Appl. Environ. Microbiol. 70, 845–849. doi: 10.1128/AEM.70.2.845-849.2004, PMID: 14766563 PMC348892

[ref25] He C. Keren R. Whittaker M. L. Farag I. F. Doudna J. A. Cate J. H. D.. (2021). Genome-resolved metagenomics reveals site-specific diversity of episymbiotic CPR bacteria and DPANN archaea in groundwater ecosystems. Nat. Microbiol. 6, 354–365. doi: 10.1038/s41564-020-00840-5, PMID: 33495623 PMC7906910

[ref26] Herrmann M. Wegner C. E. Taubert M. Geesink P. Lehmann K. Yan L.. (2019). Predominance of Cand. Patescibacteria in groundwater is caused by their preferential mobilization from soils and flourishing under oligotrophic conditions. Front. Microbiol. 10:1407. doi: 10.3389/fmicb.2019.01407, PMID: 31281301 PMC6596338

[ref27] Hosokawa S. Kuroda K. Narihiro T. Aoi Y. Ozaki N. Ohashi A.. (2021). Cometabolism of the Superphylum Patescibacteria with Anammox bacteria in a Long-term freshwater Anammox column reactor. Water 13:208. doi: 10.3390/w13020208

[ref28] Hug L. A. Baker B. J. Anantharaman K. Brown C. T. Probst A. J. Castelle C. J.. (2016). A new view of the tree of life. Nat. Microbiol. 1:16048. doi: 10.1038/nmicrobiol.2016.48 27572647

[ref29] Hugenholtz P. Pitulle C. Hershberger K. L. Pace N. R. (1998). Novel division level bacterial diversity in a Yellowstone hot spring. J. Bacteriol. 180, 366–376. doi: 10.1128/JB.180.2.366-376.1998, PMID: 9440526 PMC106892

[ref30] Jaffe A. L. Castelle C. J. Matheus Carnevali P. B. Gribaldo S. Banfield J. F. (2020). The rise of diversity in metabolic platforms across the candidate phyla radiation. BMC Biol. 18:69. doi: 10.1186/s12915-020-00804-5, PMID: 32560683 PMC7304191

[ref31] Kandlikar G. S. Gold Z. J. Cowen M. C. Meyer R. S. Freise A. C. Kraft N. J. B.. (2018). Ranacapa: An R package and shiny web app to explore environmental DNA data with exploratory statistics and interactive visualizations. F1000Research 7:1734. doi: 10.12688/f1000research.16680.1, PMID: 30613396 PMC6305237

[ref32] Kang D. D. Li F. Kirton E. Thomas A. Egan R. An H.. (2019). MetaBAT 2: an adaptive binning algorithm for robust and efficient genome reconstruction from metagenome assemblies. PeerJ 7:e7359. doi: 10.7717/peerj.7359, PMID: 31388474 PMC6662567

[ref33] Kantor R. S. Wrighton K. C. Handley K. M. Sharon I. Hug L. A. Castelle C. J.. (2013). Small genomes and sparse metabolisms of sediment-associated bacteria from four candidate phyla. MBio 4, e00708–e00713. doi: 10.1128/mBio.00708-13, PMID: 24149512 PMC3812714

[ref34] Katoh K. Standley D. M. (2013). MAFFT multiple sequence alignment software version 7: improvements in performance and usability. Mol. Biol. Evol. 30, 772–780. doi: 10.1093/molbev/mst010, PMID: 23329690 PMC3603318

[ref35] Konstantinidis K. T. Rossello-Mora R. Amann R. (2017). Uncultivated microbes in need of their own taxonomy. ISME J. 11, 2399–2406. doi: 10.1038/ismej.2017.113, PMID: 28731467 PMC5649169

[ref36] Kroeger M. E. Delmont T. O. Eren A. M. Meyer K. M. Guo J. Khan K.. (2018). New biological insights into how deforestation in Amazonia affects soil microbial communities using metagenomics and metagenome-assembled genomes. Front. Microbiol. 9:1635. doi: 10.3389/fmicb.2018.01635, PMID: 30083144 PMC6064768

[ref37] Kurtz Z. D. Müller C. L. Miraldi E. R. Littman D. R. Blaser M. J. Bonneau R. A. (2015). Sparse and compositionally robust inference of microbial ecological networks. PLoS Comput. Biol. 11:e1004226. doi: 10.1371/journal.pcbi.1004226, PMID: 25950956 PMC4423992

[ref38] Langmead B. Salzberg S. L. (2012). Fast gapped-read alignment with bowtie 2. Nat. Methods 9, 357–359. doi: 10.1038/nmeth.1923, PMID: 22388286 PMC3322381

[ref39] Lee J. Kim H. S. Jo H. Y. Kwon M. J. (2021). Revisiting soil bacterial counting methods: optimal soil storage and pretreatment methods and comparison of culture-dependent and -independent methods. PLoS One 16:e0246142. doi: 10.1371/journal.pone.0261737, PMID: 33566842 PMC7875414

[ref40] Lemos L. Manoharan L. Mendes L. Venturini A. Pylro V. Tsai S. M. (2020). Metagenome assembled-genomes reveal similar functional profiles of CPR/Patescibacteria phyla in soils. Environ. Microbiol. Rep. 12, 651–655. doi: 10.1111/1758-2229.12880, PMID: 32815317

[ref41] Lenth R. Singmann H. Love J. Buerkner P. Herve M. (2018). Emmeans: estimated marginal means, aka least-squares means. R Package Version 1.7.2.

[ref42] Letunic I. Bork P. (2019). Interactive tree of life (iTOL) v4: recent updates and new developments. Nucleic Acids Res. 47, W256–W259. doi: 10.1093/nar/gkz239, PMID: 30931475 PMC6602468

[ref43] Levy-Booth D. J. Hashimi A. Roccor R. Liu L. Y. Renneckar S. Eltis L. D.. (2021). Genomics and metatranscriptomics of biogeochemical cycling and degradation of lignin-derived aromatic compounds in thermal swamp sediment. ISME J. 15, 879–893. doi: 10.1038/s41396-020-00820-x, PMID: 33139871 PMC8027834

[ref44] Li H. Durbin R. (2009). Fast and accurate short read alignment with burrows-wheeler transform. Bioinformatics 25, 1754–1760. doi: 10.1093/bioinformatics/btp324, PMID: 19451168 PMC2705234

[ref45] Li H. Handsaker B. Wysoker A. Fennell T. Ruan J. Homer N.. (2009). The sequence alignment/map format and SAMtools. Bioinformatics 25, 2078–2079. doi: 10.1093/bioinformatics/btp352, PMID: 19505943 PMC2723002

[ref46] Li D. Liu C. M. Luo R. Sadakane K. Lam T. W. (2015). MEGAHIT: an ultra-fast single-node solution for large and complex metagenomics assembly via succinct de Bruijn graph. Bioinformatics 31, 1674–1676. doi: 10.1093/bioinformatics/btv033, PMID: 25609793

[ref47] Lombard V. Golaconda Ramulu H. Drula E. Coutinho P. M. Henrissat B. (2014). The carbohydrate-active enzymes database (CAZy) in 2013. Nucleic Acids Res. 42, D490–D495. doi: 10.1093/nar/gkt1178, PMID: 24270786 PMC3965031

[ref48] Luef B. Frischkorn K. R. Wrighton K. C. Holman H. Y. N. Birarda G. Thomas B. C.. (2015). Diverse uncultivated ultra-small bacterial cells in groundwater. Nat. Commun. 6:6372. doi: 10.1038/ncomms7372, PMID: 25721682

[ref49] Magoc T. Salzberg S. L. (2011). FLASH: fast length adjustment of short reads to improve genome assemblies. Bioinformatics 27, 2957–2963. doi: 10.1093/bioinformatics/btr507, PMID: 21903629 PMC3198573

[ref50] McCutcheon J. P. Moran N. A. (2011). Extreme genome reduction in symbiotic bacteria. Nat. Rev. Microbiol. 10, 13–26. doi: 10.1038/nrmicro2670, PMID: 22064560

[ref51] McMurdie P. J. Holmes S. (2013). Phyloseq: an R package for reproducible interactive analysis and graphics of microbiome census data. PLoS One 8:e61217. doi: 10.1371/journal.pone.0061217, PMID: 23630581 PMC3632530

[ref52] Mitchell C. C. Delaney D. Balkcom K. S. (2005). Cullars rotation: the South’s oldest continuous soil fertility experiment. Better Crops 89, 5–9.

[ref53] Mitchell C.C. Delaney D.P. Balkcom K.S. Centennial of Alabama's Cullars rotation, the South's oldest, continuous soil fertility experiment. (2011). Alabama’s Agricultural Experiment Station (Auburn University).

[ref54] Nakai R. (2020). Size matters: ultra-small and filterable microorganisms in the environment. Microbes Environ. 35:ME20025. doi: 10.1264/jsme2.ME20025, PMID: 32493880 PMC7308576

[ref55] Nicolas A. M. Jaffe A. L. Nuccio E. E. Taga M. E. Firestone M. K. Banfield J. F.. (2021). Soil candidate phyla radiation bacteria encode components of aerobic metabolism and co-occur with Nanoarchaea in the rare biosphere of rhizosphere grassland communities. mSystems 6:e0120520. doi: 10.1128/mSystems.01205-20, PMID: 34402646 PMC8407418

[ref56] Oksanen J. Blanchet F. G. Friendly M. Kindt R. Legendre P. McGlinn D.. (2013). Package ‘vegan’. Community ecology package, version 2, 1–295.

[ref57] Olm M. R. Brown C. T. Brooks B. Banfield J. F. (2017). dRep: a tool for fast and accurate genomic comparisons that enables improved genome recovery from metagenomes through de-replication. ISME J. 11, 2864–2868. doi: 10.1038/ismej.2017.126, PMID: 28742071 PMC5702732

[ref58] Parks D. H. Chuvochina M. Chaumeil P. A. Rinke C. Mussig A. J. Hugenholtz P. (2020). A complete domain-to-species taxonomy for bacteria and archaea. Nat. Biotechnol. 38, 1079–1086. doi: 10.1038/s41587-020-0501-8, PMID: 32341564

[ref59] Parks D. H. Chuvochina M. Rinke C. Mussig A. J. Chaumeil P. A. Hugenholtz P. (2022). GTDB: an ongoing census of bacterial and archaeal diversity through a phylogenetically consistent, rank normalized and complete genome-based taxonomy. Nucleic Acids Res. 50, D785–D794. doi: 10.1093/nar/gkab776, PMID: 34520557 PMC8728215

[ref60] Parks D. H. Chuvochina M. Waite D. W. Rinke C. Skarshewski A. Chaumeil P. A.. (2018). A standardized bacterial taxonomy based on genome phylogeny substantially revises the tree of life. Nat. Biotechnol. 36, 996–1004. doi: 10.1038/nbt.4229, PMID: 30148503

[ref61] Parks D. H. Imelfort M. Skennerton C. T. Hugenholtz P. Tyson G. W. (2015). CheckM: assessing the quality of microbial genomes recovered from isolates, single cells, and metagenomes. Genome Res. 25, 1043–1055. doi: 10.1101/gr.186072.114, PMID: 25977477 PMC4484387

[ref62] Parks D. H. Rinke C. Chuvochina M. Chaumeil P. A. Woodcroft B. J. Evans P. N.. (2017). Recovery of nearly 8,000 metagenome-assembled genomes substantially expands the tree of life. Nat. Microbiol. 2, 1533–1542. doi: 10.1038/s41564-017-0012-7, PMID: 28894102

[ref63] Paulson J. N. Stine O. C. Bravo H. C. Pop M. (2013). Differential abundance analysis for microbial marker-gene surveys. Nat. Methods 10, 1200–1202. doi: 10.1038/nmeth.2658, PMID: 24076764 PMC4010126

[ref64] Portillo M. C. Leff J. W. Lauber C. L. Fierer N. (2013). Cell size distributions of soil bacterial and archaeal taxa. Appl. Environ. Microbiol. 79, 7610–7617. doi: 10.1128/AEM.02710-13, PMID: 24077710 PMC3837822

[ref65] Pruesse E. Peplies J. Glockner F. O. (2012). SINA: accurate high-throughput multiple sequence alignment of ribosomal RNA genes. Bioinformatics 28, 1823–1829. doi: 10.1093/bioinformatics/bts252, PMID: 22556368 PMC3389763

[ref66] Quast C. Pruesse E. Yilmaz P. Gerken J. Schweer T. Yarza P.. (2012). The SILVA ribosomal RNA gene database project: improved data processing and web-based tools. Nucleic Acids Res. 41, D590–D596. doi: 10.1093/nar/gks1219, PMID: 23193283 PMC3531112

[ref67] Rinke C. Schwientek P. Sczyrba A. Ivanova N. N. Anderson I. J. Cheng J. F.. (2013). Insights into the phylogeny and coding potential of microbial dark matter. Nature 499, 431–437. doi: 10.1038/nature12352, PMID: 23851394

[ref68] Sanchez R. Serra F. Tarraga J. Medina I. Carbonell J. Pulido L.. (2011). Phylemon 2.0: a suite of web-tools for molecular evolution, phylogenetics, phylogenomics and hypotheses testing. Nucleic Acids Res. 39, W470–W474. doi: 10.1093/nar/gkr408, PMID: 21646336 PMC3125789

[ref69] Santana-Pereira A. L. R. Sandoval-Powers M. Monsma S. Zhou J. Santos S. R. Mead D. A.. (2020). Discovery of novel biosynthetic gene cluster diversity from a soil metagenomic library. Front. Microbiol. 11:585398. doi: 10.3389/fmicb.2020.585398, PMID: 33365020 PMC7750434

[ref70] Schloss P. D. Westcott S. L. Ryabin T. Hall J. R. Hartmann M. Hollister E. B.. (2009). Introducing mothur: open-source, platform-independent, community-supported software for describing and comparing microbial communities. Appl. Environ. Microbiol. 75, 7537–7541. doi: 10.1128/AEM.01541-09, PMID: 19801464 PMC2786419

[ref71] Segata N. Izard J. Waldron L. Gevers D. Miropolsky L. Garrett W. S.. (2011). Metagenomic biomarker discovery and explanation. Genome Biol. 12:R60. doi: 10.1186/gb-2011-12-6-r60, PMID: 21702898 PMC3218848

[ref72] Shaffer M. Borton M. A. McGivern B. B. Zayed A. A. la Rosa S. L. Solden L. M.. (2020). DRAM for distilling microbial metabolism to automate the curation of microbiome function. Nucleic Acids Res. 48, 8883–8900. doi: 10.1093/nar/gkaa621, PMID: 32766782 PMC7498326

[ref73] Sharon I. Banfield J. F. (2013). Microbiology. Genomes from metagenomics. Science 342, 1057–1058. doi: 10.1126/science.1247023, PMID: 24288324

[ref74] Sharrar A. M. Crits-Christoph A. Méheust R. Diamond S. Starr E. P. Banfield J. F. (2020). Bacterial secondary metabolite biosynthetic potential in soil varies with phylum, depth, and vegetation type. MBio 11:e00416-20. doi: 10.1128/mBio.00416-20, PMID: 32546614 PMC7298704

[ref75] Sieber C. M. K. Probst A. J. Sharrar A. Thomas B. C. Hess M. Tringe S. G.. (2018). Recovery of genomes from metagenomes via a dereplication, aggregation and scoring strategy. Nat. Microbiol. 3, 836–843. doi: 10.1038/s41564-018-0171-1, PMID: 29807988 PMC6786971

[ref76] Smoot M. E. Ono K. Ruscheinski J. Wang P. L. Ideker T. (2011). Cytoscape 2.8: new features for data integration and network visualization. Bioinformatics 27, 431–432. doi: 10.1093/bioinformatics/btq675, PMID: 21149340 PMC3031041

[ref77] Stamatakis A. (2014). RAxML version 8: a tool for phylogenetic analysis and post-analysis of large phylogenies. Bioinformatics 30, 1312–1313. doi: 10.1093/bioinformatics/btu033, PMID: 24451623 PMC3998144

[ref78] Starr E. P. Shi S. Blazewicz S. J. Probst A. J. Herman D. J. Firestone M. K.. (2018). Stable isotope informed genome-resolved metagenomics reveals that Saccharibacteria utilize microbially-processed plant-derived carbon. Microbiome 6:122. doi: 10.1186/s40168-018-0499-z, PMID: 29970182 PMC6031116

[ref79] Taubert M. Stahly J. Kolb S. Kusel K. (2019). Divergent microbial communities in groundwater and overlying soils exhibit functional redundancy for plant-polysaccharide degradation. PLoS One 14:e0212937. doi: 10.1371/journal.pone.0212937, PMID: 30865693 PMC6415789

[ref80] Torsvik V. Ovreas L. (2002). Microbial diversity and function in soil: from genes to ecosystems. Curr. Opin. Microbiol. 5, 240–245. doi: 10.1016/S1369-5274(02)00324-7, PMID: 12057676

[ref81] Wagner D. Kobabe S. Liebner S. (2009). Bacterial community structure and carbon turnover in permafrost-affected soils of the Lena Delta, northeastern Siberia. Can. J. Microbiol. 55, 73–83. doi: 10.1139/W08-121, PMID: 19190703

[ref82] Woodcroft B. J. Singleton C. M. Boyd J. A. Evans P. N. Emerson J. B. Zayed A. A. F.. (2018). Genome-centric view of carbon processing in thawing permafrost. Nature 560, 49–54. doi: 10.1038/s41586-018-0338-1, PMID: 30013118

[ref83] Wrighton K. C. Castelle C. J. Varaljay V. A. Satagopan S. Brown C. T. Wilkins M. J.. (2016). RubisCO of a nucleoside pathway known from archaea is found in diverse uncultivated phyla in bacteria. ISME J. 10, 2702–2714. doi: 10.1038/ismej.2016.53, PMID: 27137126 PMC5113843

[ref84] Wrighton K. C. Castelle C. J. Wilkins M. J. Hug L. A. Sharon I. Thomas B. C.. (2014). Metabolic interdependencies between phylogenetically novel fermenters and respiratory organisms in an unconfined aquifer. ISME J. 8, 1452–1463. doi: 10.1038/ismej.2013.249, PMID: 24621521 PMC4069391

[ref85] Wrighton K. C. Thomas B. C. Sharon I. Miller C. S. Castelle C. J. VerBerkmoes N. C.. (2012). Fermentation, hydrogen, and sulfur metabolism in multiple uncultivated bacterial phyla. Science 337, 1661–1665. doi: 10.1126/science.1224041, PMID: 23019650

[ref86] Wu Y. W. Simmons B. A. Singer S. W. (2016). MaxBin 2.0: an automated binning algorithm to recover genomes from multiple metagenomic datasets. Bioinformatics 32, 605–607. doi: 10.1093/bioinformatics/btv638, PMID: 26515820

[ref87] Yeates C. Holmes A. J. Gillings M. R. (2000). Novel forms of ring-hydroxylating dioxygenases are widespread in pristine and contaminated soils. Environ. Microbiol. 2, 644–653. doi: 10.1046/j.1462-2920.2000.00147.x, PMID: 11214797

[ref88] Yuan Q. Xueyu P. Wei J. Lianqing C. Zhilin Y. (2018). Comparison of four extraction methods of soil microbiome in poplar plantation. Scientia Silvae Sinicae 54, 169–176.

[ref89] Zhao C. Fu S. Mathew R. P. Lawrence K. S. Feng Y. (2015). Soil microbial community structure and activity in a 100-year-old fertilization and crop rotation experiment. J. Plant Ecol. 8, rtv007–rtv632. doi: 10.1093/jpe/rtv007

