# Supplementary material for: Influence of soil nutrients on the presence and distribution of CPR bacteria in a long-term crop rotation experiment
Source: Front Microbiol. 2023 Jul 27;14:1114548. doi: 10.3389/fmicb.2023.1114548 (PMC10413278; doi:10.3389/fmicb.2023.1114548)
Supplement: Supplementary file 1 [file Data_Sheet_1.pdf]

Influence of soil nutrients on the presence and distribution of CPR bacteria in a long-term crop rotation experiment

Alinne L. R. Santana-Pereira[1], Francesco S. Moen[1], Beatrice Severance[1], Mark R. Liles[1*]
[1]Auburn University, Department of Biological Sciences, Auburn, Alabama, USA
***Corresponding Author:** *Mark R. Liles* lilesma@auburn.edu

Keywords: candidate phyla radiation, metagenome-assembled genomes, metabolism, phylogenetic diversity, soil ecology

Figures and Legends

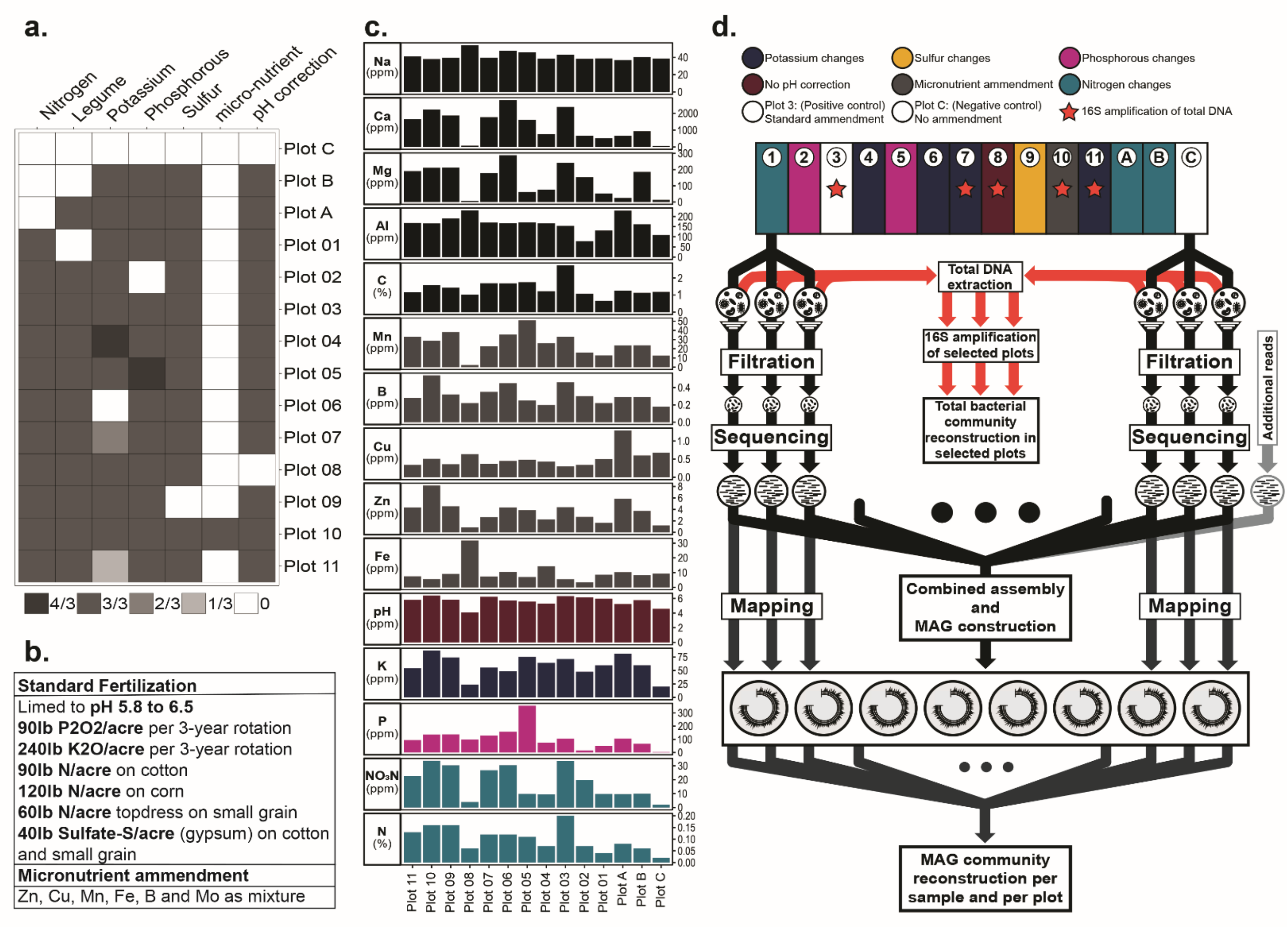

Figure 1. Representation of Cullars Rotation plots and Fertilization treatments. **a.** Schematic representation of Cullars Rotation Plot layout and Soil treatments administered. pH correction: pH correction with lime. Fractions represent the amount added to the plot with Standard Fertilization as reference (3/3). **b.** Table with the Standard Fertilization treatments applied to each plot (Mitchell *et al.*, 2005). c. Average soil nutrient parameters from sampled bulk soil. **d.** Overview of the methods employed to reconstruct the MAGs from the filtration enrichment (mini-metagenome), reconstruct the MAG community and reconstruct the total bacterial community using 16S amplification from soil total DNA extraction.

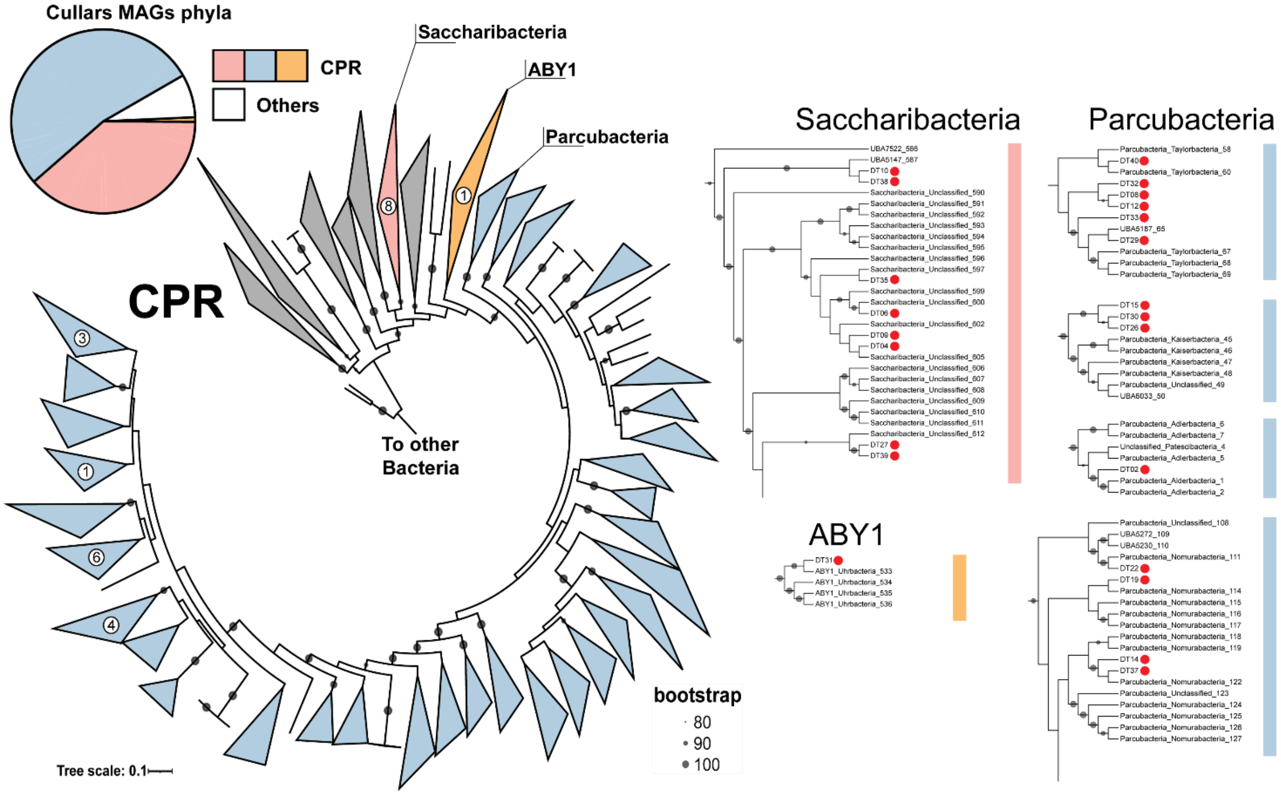

Figure 2. Classification of all Cullars MAGs and the proportion of each phyla. *Total MAGs does not include MAGs filtered out by the completeness, strain heterogeneity and contamination thresholds. Concatenated maximum likelihood tree of 13 phylogenetic marker genes from Cullars CPR and 920 reference genomes allowed further classification of CPR MAGs. MAGs were classified as Saccharibacteria (Saccharimonadia), ABY1 and Parcubacteria (Paceibacteria). The Parcubacteria group is expanded in the tree due to its intrinsic diversity. Zoomed in regions offers context to MAGs placement. Red dots denote Cullars MAGs.

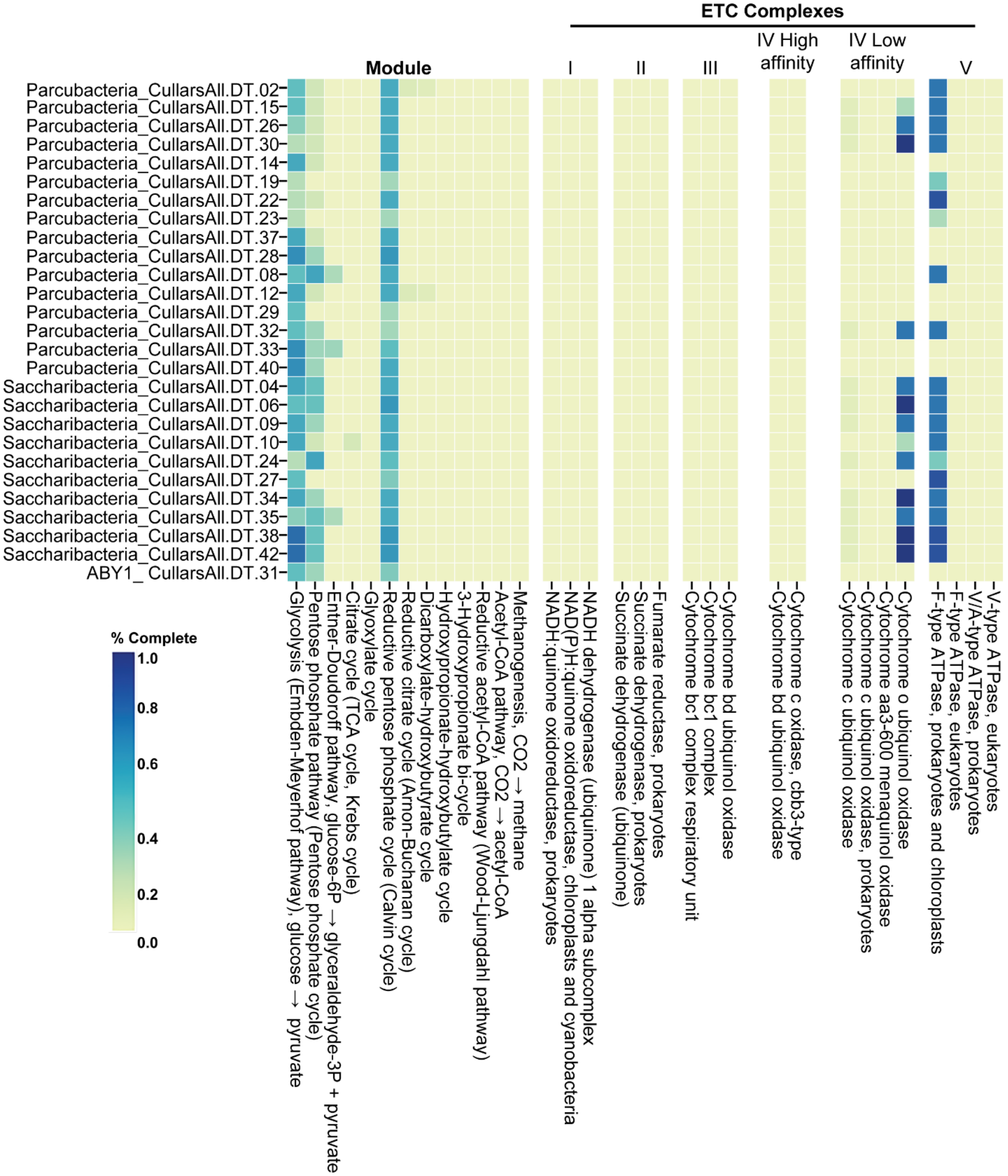

Figure 3. Energetic metabolic potential of Cullars MAGs. % Complete: level of completeness of the pathway, measured as presence of genes coding for necessary enzymes to carry out each step.

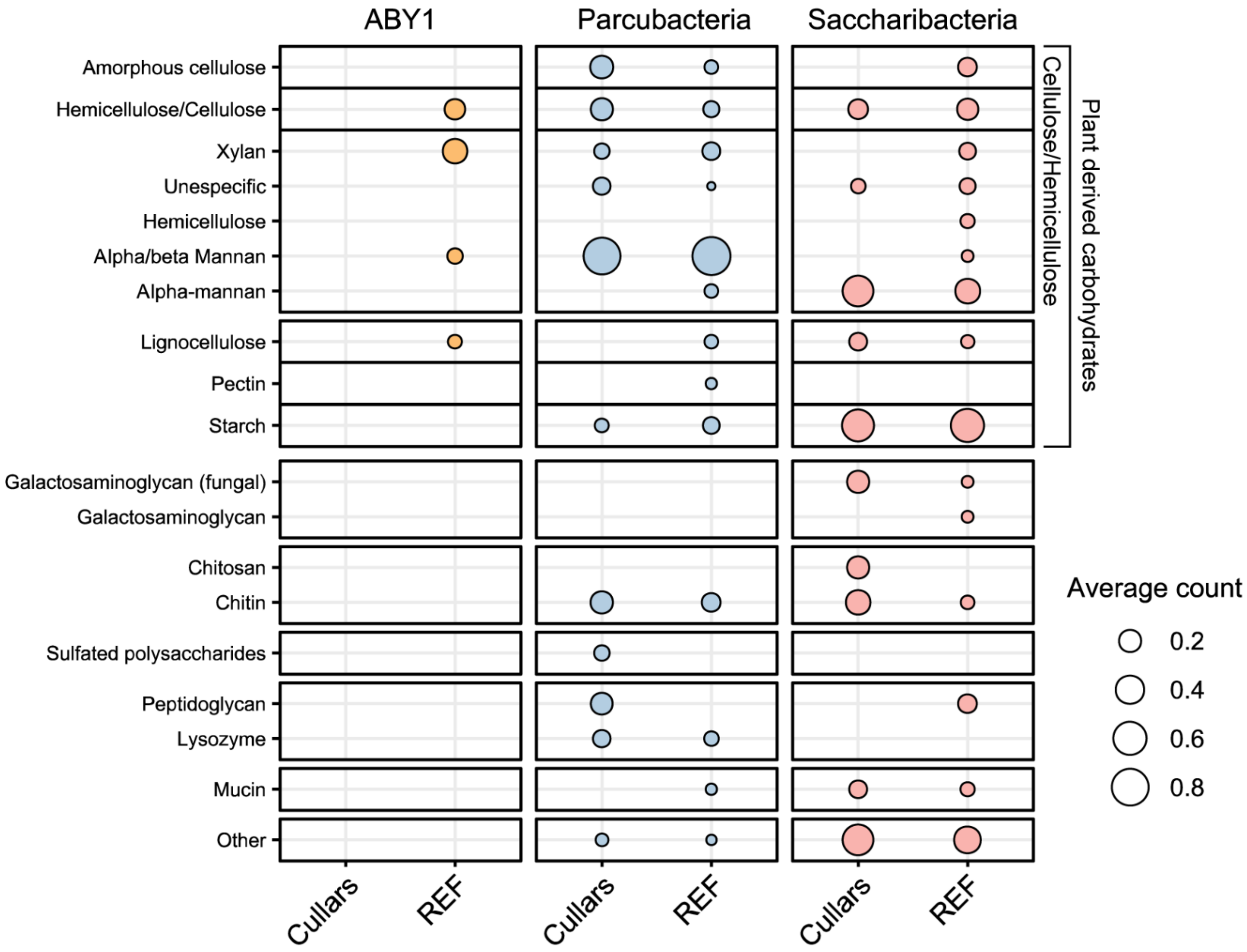

Figure 4. Carbohydrate degradation potential of Cullars CPR MAGs and their references. Hits correspond to GH genes identified against the CAZy database, which was also used to obtain substract information. Circles represent the averaged hits for references and Cullars MAGs per substrate category.

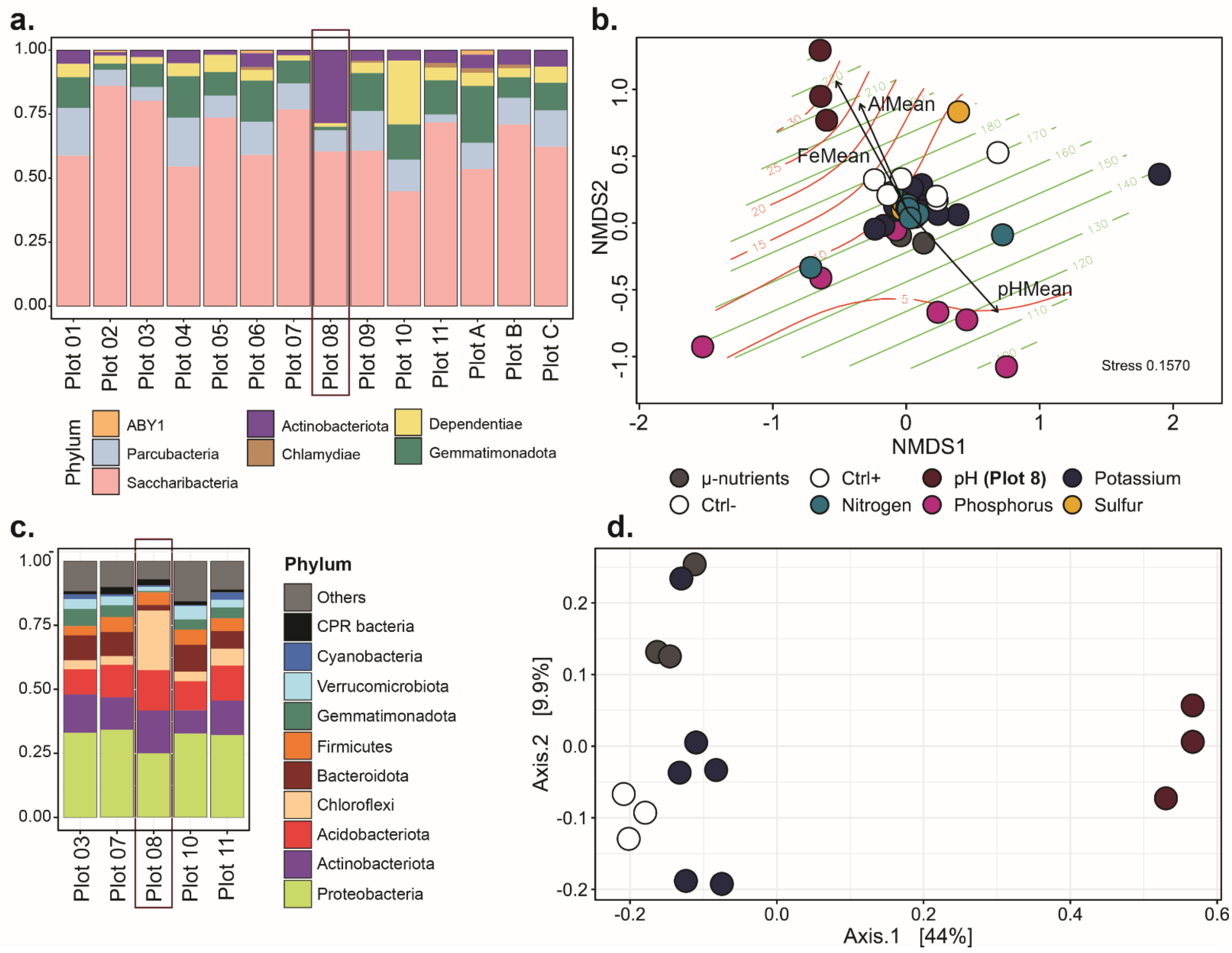

Figure 5. a. MAG community reconstruction from Cullars soil at different plots corresponding MAG average relative abundance, ranked by Phyla. **b.** NMDS ordination of all sample communities with Bray-Curtis distances. Colors emphasize the varying nutrients and pH on the plots. Ctrl +: Plot 3 (Standard Fertilization); Ctrl -: Plot C (No fertilization or amendment). Green lines: Aluminum levels; Red lines: Iron levels. Vectors are soil parameters significantly related to community changes ($p < 0.05$). Emphasis on Plot 8 samples, which are pulled in the direction of the highest Fe and Al concentrations. **c.** Overall community reconstruction from Cullars soil of selected Cullars plots using 16S amplification. **d.** Ordination plot by PCA of all sample communities with Bray-Curtis distances.

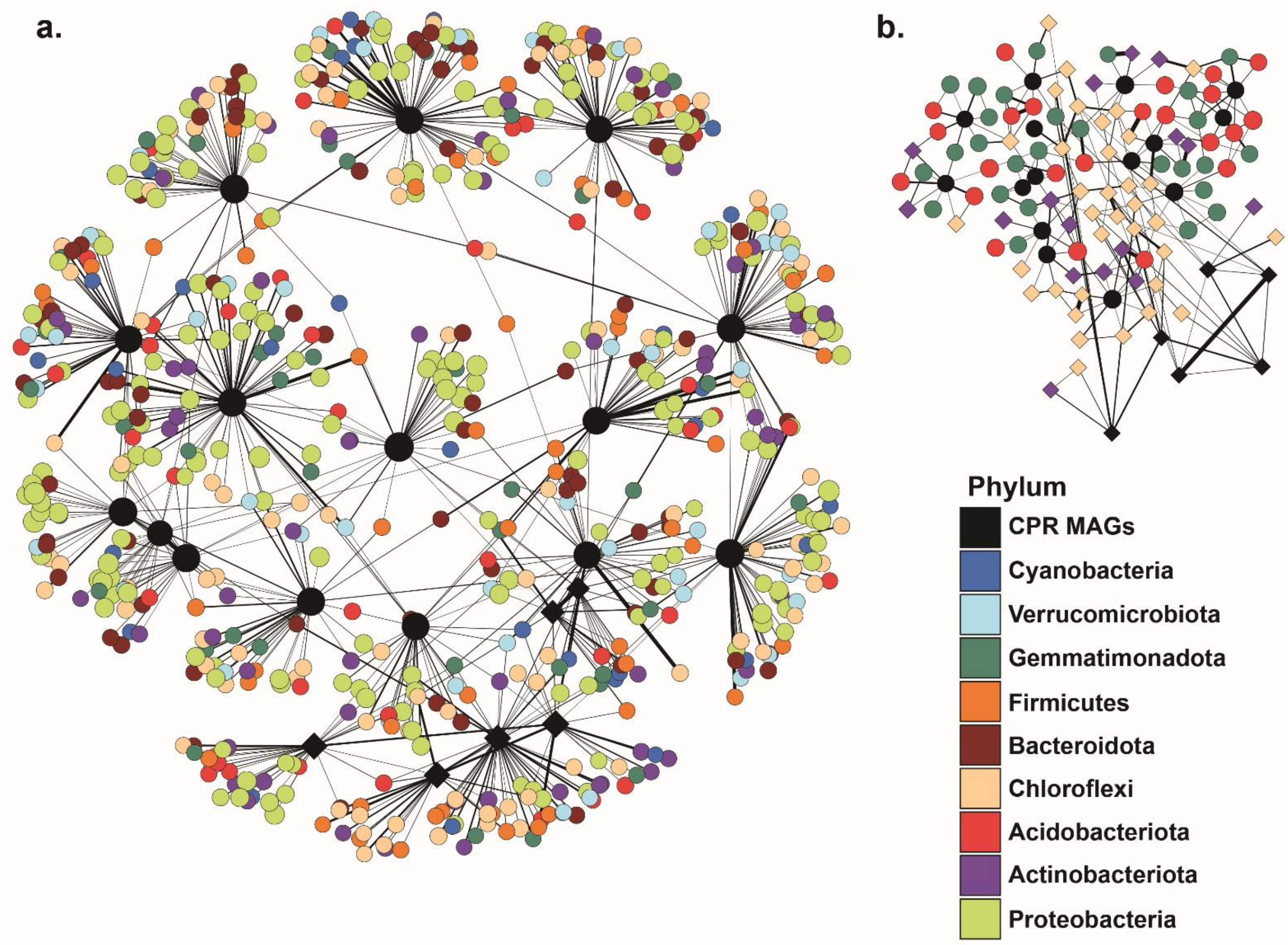

Figure 06. a. Network analysis filtered by all the OTUS belonging to the main phyla from the overall microbiota that interacts positively with the Cullars MAGs. Line thickness corresponds to correlation weight **b.** Network analysis filtered by all the OTUS belonging to the plot biomarkers (defined by LEfS analysis) that interacts positively with the Cullars MAGs. Diamond shaped nodes: Prominent MAGs and biomarkers in Plot 08.
